# Truncated conjugation in fused heterocycle-based conducting polymers: when greater planarity does not enhance conjugation†

**DOI:** 10.1039/d2sc06271b

**Published:** 2022-12-07

**Authors:** Jose Manuel Marin-Beloqui, Sandra Gómez, Hristo Ivov Gonev, Marc Comí, Mohammed Al-Hashimi, Tracey M. Clarke

**Affiliations:** a Department of Chemistry, University College London Christopher Ingold Building London WC1H 0AJ UK tracey.clarke@ucl.ac.uk; b Department of Physical Chemistry, University of Malaga Blvrd Louis Pasteur 31 29010 Malaga Spain jm.marinbeloqui@uma.es; c Department of Physical Chemistry, University of Salamanca Caidos Sq. 37008 Salamanca Spain; d Department of Chemistry, Texas A&M University at Qatar Education City, P. O. Box 23874 Doha Qatar

## Abstract

One of the main assumptions in the design of new conjugated polymer materials for their use in organic electronics is that higher coplanarity leads to greater conjugation along the polymer backbone. Conventionally, a more planar monomer structure induces a larger backbone coplanarity, thus leading to a greater overlap of the carbon π-orbitals and therefore a higher degree of π-electron delocalisation. However, here we present a case that counters the validity of this assumption. Different diselenophene-based polymers were studied where one polymer possesses two selenophene rings fused together to create a more rigid, planar structure. The effects of this greater polymer coplanarity were examined using Raman spectroscopy and theoretical calculations. Raman spectra showed a large difference between the vibrational modes of the fused and unfused polymers, indicating very different electronic structures. Resonance Raman spectroscopy confirmed the rigidity of the fused selenophene polymer and also revealed, by studying the excitation profiles of the different bands, the presence of two shorter, uncoupled conjugation pathways. Supported by Density Functional Theory (DFT) calculations, we have demonstrated that the reason for this lack of conjugation is a distortion of the selenophene rings due to the induced planarity, forming a new truncated conjugation pathway through the selenophene β-position and bypassing the beneficial α-position. This effect was studied using DFT in an ample range of derivatives, where substitution of the selenium atom with other heteroatoms still maintained the same unconventional conjugation–planarity relationship, confirming the generality of this phenomenon. This work establishes an important structure–property relationship for conjugated polymers that will help rational design of more efficient organic electronics materials.

## Abstract 2

A Raman spectroscopy and theoretical study to find an unconventional structure-property relationship. A larger planarity forms an alternative truncated conjugation pathway that hinders the beneficial a-conjugation selenophene pathway.

## Graphical abstract

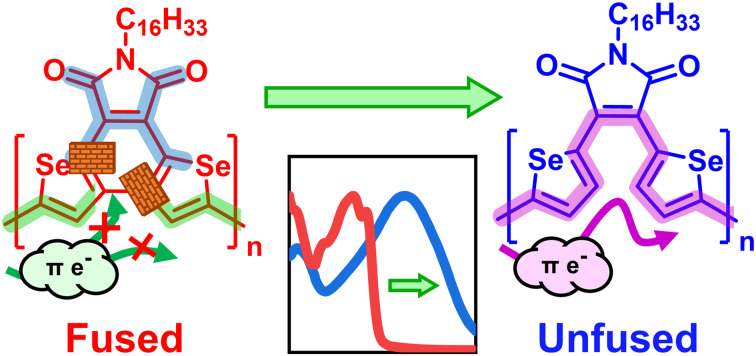

## Introduction

Organic conjugated polymers have gained considerable attention due to their wide range of optoelectronic applications and accompanying potential low cost of fabrication and manufacture. One of the key features of conjugated polymers is the ability to fine-tune their properties through structural modification. Therefore, the ability to establish causal relationships between molecular structure and electronic properties is a pivotal point in organic electronics. Structure–property relationships enable rational design of new materials for a specific purpose, avoiding costly trial-and-error methods.^1–3^

In general, planar polymers are desired in organic optoelectronics. At the molecular level, greater planarity is associated with better overlap of the carbon p-orbitals and therefore a higher π-conjugation along the polymer backbone. Planar polymer structures also usually allow better π–π stacking in the solid phase due to enhanced overlap between the π-electron clouds of adjacent polymer chains. Better π–π stacking between polymer chains stabilises the presence of excited states, enhancing the lifetimes and mobilities of these species. This typically increases the suitability of these materials for organic devices such as solar cells, light-emitting diodes, and field-effect transistors.^4–6^ In fact, a wide range of examples can be found in literature where fusing the heterocycles of the polymer leads to larger planarity and greater backbone π-conjugation.^4,7–11^ Synthetic chemists also pursue planarisation by enhancement of non-covalent interactions.^12–16^ The extensive literature where the planarisation strategy has been followed clearly proves how established heterocycle fusing is in the field.

**Fig. 1:**
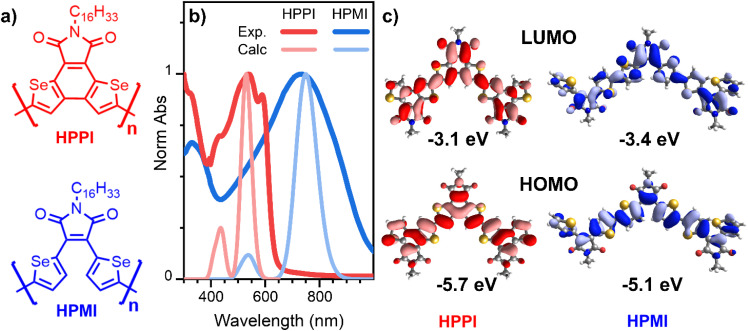
(a) Molecular structures of HPPI and HPMI polymers. (b) Comparison of experimental (darker lines) and calculated trimer (lighter lines) normalised ground state absorbance of HPPI (red) and HPMI (blue) films. (c) Calculated HOMO (bottom) and LUMO (top) of HPPI (left) and HPMI (right) trimers. Calculations were carried out at B3LYP/6-31G** level.

One of the clearest outcomes of most heterocycle fusing is that the greater π-conjugation along the backbone leads to a decrease of the optical bandgap. A longer effective π-conjugation leads to the delocalisation of the molecular orbitals, raising the HOMO (highest occupied molecular orbital) energy and lowering the LUMO (lowest unoccupied molecular orbital) energy. Hence, the optical absorption of materials with larger effective conjugation will be displaced to longer wavelengths. This is particularly clear in the case of oligomers, where a larger number of monomers pushes the absorbance to longer wavelengths.^17^ In our previous work, we studied two diselenophene-based polymers (Fig. 1a) for their use in organic transistors, where a fusing strategy was followed.^18^ One polymer possesses two separate selenophene rings in its maleimide monomer unit (HPMI), whereas the selenophene rings are fused to create a rigid selenobenzene–maleimide moiety in the second polymer (HPPI) to induce greater backbone co-planarity. Intriguingly, the fused selenophene polymer (HPPI) led to a more blue-shifted absorbance than its unfused counterpart (HPMI), which is usually an indication of lower effective conjugation.

In this work we have studied the origin of this unexpected observation in HPMI and HPPI. The electronic properties of these materials were studied with a series of theoretical and experimental techniques in order to find the origin of HPPI's unusually blue-shifted absorbance. Specifically, Raman spectroscopy was used for this analysis because it is particularly useful to study the electronic structure of conjugated organic materials. Double bonds involved in the π-conjugation along a polymer backbone have an intrinsic high polarisable electron density along the bond axis, which causes a large Raman intensity in the vibrations associated with these double bonds.

We have found that fusing the selenophene rings, and thus increasing planarity, distorted the selenophene rings and created a new conjugation pathway through the selenophene β-position, bypassing the beneficial α-position.^19,20^ In addition, through a series of calculations it was found that this ‘truncated conjugation’ effect was a general phenomenon for a series of phenanthrene-like derivatives, independent of the heteroatom present in the five-atom ring.

This phenomenon, based purely on the molecular structure of the material, highlights the importance of this work and its implications for further understanding of the structure–function relationships in organic electronics. Establishing these relationships will be of upmost importance in the field of organic electronics, for example explaining the low efficiency of HPPI transistors.^18^ In addition, the rational elucidation of the large changes in electronic characteristics upon photochemical cyclisation reactions is of great interest in the field of molecular photoswitches.^21–23^

## Results

Fig. 1b shows the experimental normalised ground state absorbance of HPPI and HPMI films. The HPPI film shows marked vibronic structure with an absorbance maximum at 540 nm. On the other hand, unfused HPMI shows a featureless broader band centred at 740 nm. The clearer vibronic structure and lower full width at half maximum (FWHM, 0.77 eV) of the HPPI absorption indicates a larger structural rigidity and thus less inhomogeneous broadening in comparison with HPMI (0.98 eV FWHM), as imparted by the fusion of the two selenophene rings in HPPI.^24^ However, despite this rigidity and lack of conformational variation, the fused ring polymer HPPI absorbance is considerably blue-shifted by 0.62 eV in comparison to the unfused polymer. GIWAXS measurements previously published indicate that both as-cast polymer films were amorphous.^18^ The similarity of the absorption maximum positions in both solution and film (Fig. S1†) – and the pronounced blue-shift compared to HPMI, even in solution – suggest an intrinsic origin to HPPI's larger optical band gap.

One possible origin for this blue-shifted absorbance could be the effective length of the polymer chains. However, both polymers have similar average molecular mass (see Table S1†). Polydispersity may arise as another explanation for this unusual absorbance shift. A larger number of polymers with different chain sizes produces an extensive number of polymer conformations which can induce solid state defects, causing an absorbance red-shift.^25,26^ However, HPMI has both redder absorbance and lower polydispersity (1.4) compared to HPPI (1.9). Therefore, the unusual behaviour in the absorbance cannot be explained by differences in synthesis and polymer chain length.

**Fig. 2:**
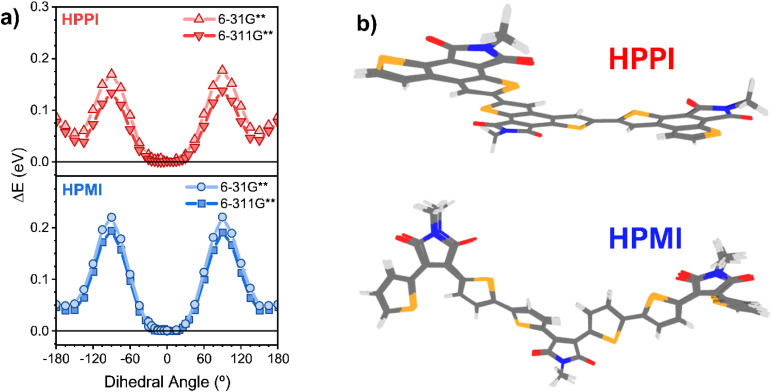
(a) Relaxed potential energy scan around the Se–C–C–Se dihedral bond performed with B3LYP/6-31G** and B3LYP/6-311G**. The *y* axis, Δ*E*, is the energy relative to the global minimum. (b) Superimposed ground state optimised geometries calculated at the levels of theory B3LYP/6-31G**, B3LYP/6-311G**, CAM-B3LYP/6-31G**, CAM-B3LYP/6-311G** and ωB97X-D3/6-31G** of HPPI (top) and HPMI (bottom) trimers.

To elucidate the unexpected relative absorbance seen in these two materials, density functional theory (DFT) calculations have been carried out. An alkyl-truncated trimer model was used to simplify the calculations. Calculations of the dimer and the tetramer were also performed to confirm the suitability of the chosen model size (Fig. S2†). Since the connection – or lack thereof – between coplanarity and conjugation is at the core of this manuscript, we start with a direct assessment of the Se–C–C–Se inter-monomeric dihedral angles (with atoms 12–13–14–15 as an example, Scheme S1†). We perform relaxed potential energy scans (B3LYP/6-31G** and B3LYP/6-311G**) where 0° is defined as the fully planar geometry with the Se atoms in a *trans* configuration (Fig. 2a). The potential energy scans show that both polymer models have a broad global minimum around 0°, with HPMI being closer to 15°.

Given the broadness of these minima, the fully optimised geometries of the trimer models were calculated using a diverse set of functionals (B3LYP, and long-range corrected CAM-B3LYP and ωB97X-D3) and basis sets (6-31G** and 6-311G**). This is particularly important to ensure that any artificial overdelocalisation of the wave function is avoided, as has been previously observed using the B3LYP functional.^27^ The superposed results of these geometry optimisation calculations can be found in Fig. 2b. Intriguingly, all methods/basis set combinations gave almost identical structures for each material, indicating the validity of the results presented in the rest of the manuscript. The calculated HPPI trimer shows a completely planar monomer structure while the HPMI monomers are non-planar and form an 11.6° dihedral angle between the selenophene and maleimide moieties. Even more importantly for our purposes, the dihedral Se–C–C–Se angle between adjacent monomer units is approximately 0° for HPPI (Table S2†), indicating a fully coplanar structure. Conversely, the inter-monomeric dihedral angle is larger for HPMI (∼15°), irrespective of method/basis set, thereby indicating a reduced coplanarity. This ∼15° variation in the backbone torsion therefore cannot explain such large changes in bandgap (0.6 eV variation). Since a strong planarity is calculated *in vacuo* for HPPI, this planarity is also expected to be maintained in solid state, as in solid state there is an additional contribution from intermolecular packing.^28–30^

Calculated absorbance spectra using TD-B3LYP/6-31G** (Fig. 1b and S3†) show a main band at 530 and 750 nm for HPPI and HPMI, respectively, corresponding to the HOMO–LUMO transition. Interestingly, the magnitude of the red-shift from HPPI to HPMI is almost identical in the experimental and calculated data (0.62 and 0.69 eV, respectively). The unconventional relationship between the experimentally observed bandgaps was thus reproduced by the calculations: the more planar material possesses the larger bandgap. This also confirms that the origin of this phenomenon is purely electronic since solid-state interactions are not considered in these calculations. Fig. 1c shows the calculated HOMO and LUMO for both materials. The HOMO energy level of HPMI showed a long concatenation of bonding orbitals along the whole HPMI backbone, including the maleimide double bond. This concatenation of bonding orbitals suggests an effective π-conjugation along the whole backbone. However, in the HOMO of HPPI polymer, this conjugation is not followed. There is a considerable shift of orbital density from α-β CC to the β-β CC on the terminal rings, including a contribution from the Se atoms. This indicates a large difference in the electronic distribution of both homopolymers, a possible origin of the unconventional bandgap difference between these π-conjugated materials.

It is vital to experimentally confirm the enhanced coplanarity for HPPI relative to HPMI. If the reverse were true, this would pose a simple explanation for the unconventional absorbance relationship seen for these materials. To obtain more information on the electronic structure of these materials, FT-Raman and resonance Raman spectroscopy have been performed on the polymer films. Raman spectroscopy has been extensively used to assess planarity, crystallinity, and morphology of organic photovoltaic materials, owing to its very high degree of structural and conformational sensitivity.^31–33^ Furthermore, the resonance Raman effect enables correlations to be made between the molecular vibrations and electronic structure. These aspects make it the ideal experimental technique for our purposes, enabling both the planarity and the conjugation to be probed simultaneously.

**Fig. 3:**
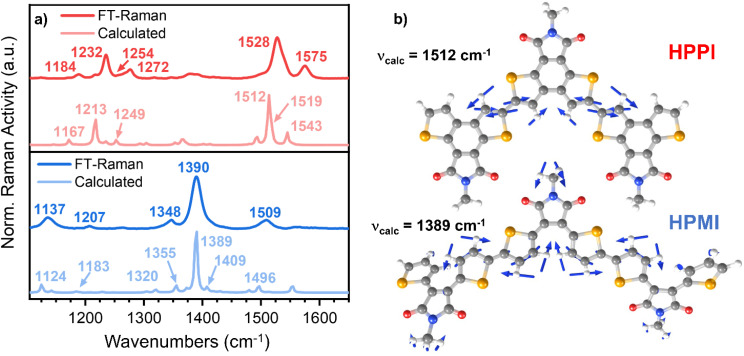
(a) Comparison of the calculated (B3LYP/6-31G**l) (lighter lines) and experimental (darker lines) FT-Raman spectra for HPPI (top) and HPMI (bottom) films. (b) Eigenvectors of the vibrational modes corresponding to the most intense Raman band for HPPI and HPMI (experimentally seen at 1528 and 1390 cm^−1^, respectively).

Fig. 3a shows the results of the FT-Raman characterisation of HPPI and HPMI. Quantum chemistry calculations on trimer models using B3LYP/6-31G**, carefully benchmarked against different functionals and basis sets (as discussed earlier) were performed to help elucidate which vibrational modes are associated with each Raman band. The assignments were made by comparing the band intensities and similarities in frequency displacements between calculated and FT-Raman spectra. Frequency differences between calculated and experimental bands were maintained between 5 and 30 cm^−1^. A full and detailed explanation of the Raman band assignments can be found in the ESI.†

Interestingly, HPPI and HPMI showed very different Raman spectra. Their most intense bands were associated with different vibrational modes (Fig. 3b): the 1528 cm^−1^ HPPI band corresponded to the central selenophene C=C stretches while the most intense 1390 cm^−1^ HPMI band was assigned to C–C stretches along the polymer backbone. It is quite unusual for a C–C stretch to be the most intense band in a Raman spectrum, however it should also be noted that this mode is localised on the central rings of the trimer model and the calculated backbone bond lengths in this region are all roughly equivalent (*vide infra*), implying significant delocalisation of the π-electrons. The large difference in the nature and the position of the vibrational modes associated with the highest intensity vibrational band between HPMI and HPPI indicates a large change in the electronic structure of the materials upon fusing the rings. This agrees with the large differences seen in the π-electron density of the calculated HOMO levels (Fig. 1c).

Another remarkable difference between HPPI and HPMI Raman spectra are the bands associated with the maleimide vibrational modes. The maleimide C–C stretch (bonds C_5_–C_6_ and C_8_–C_9_, atom labelling shown in Scheme S1†) undergoes a considerable upshift from HPMI (experimentally seen at 1137 cm^−1^, calculated at 1124 cm^−1^) to HPPI (experimentally seen at 1232 cm^−1^ calculated at 1213 cm^−1^). This large upshift suggests an increased force constant and thus substantially more double-bond character of the maleimide single bonds. This implies that the fusion of the selenophene rings leads to a larger contribution of the HPPI maleimide moiety in the polymer conjugation.

**Fig. 4:**
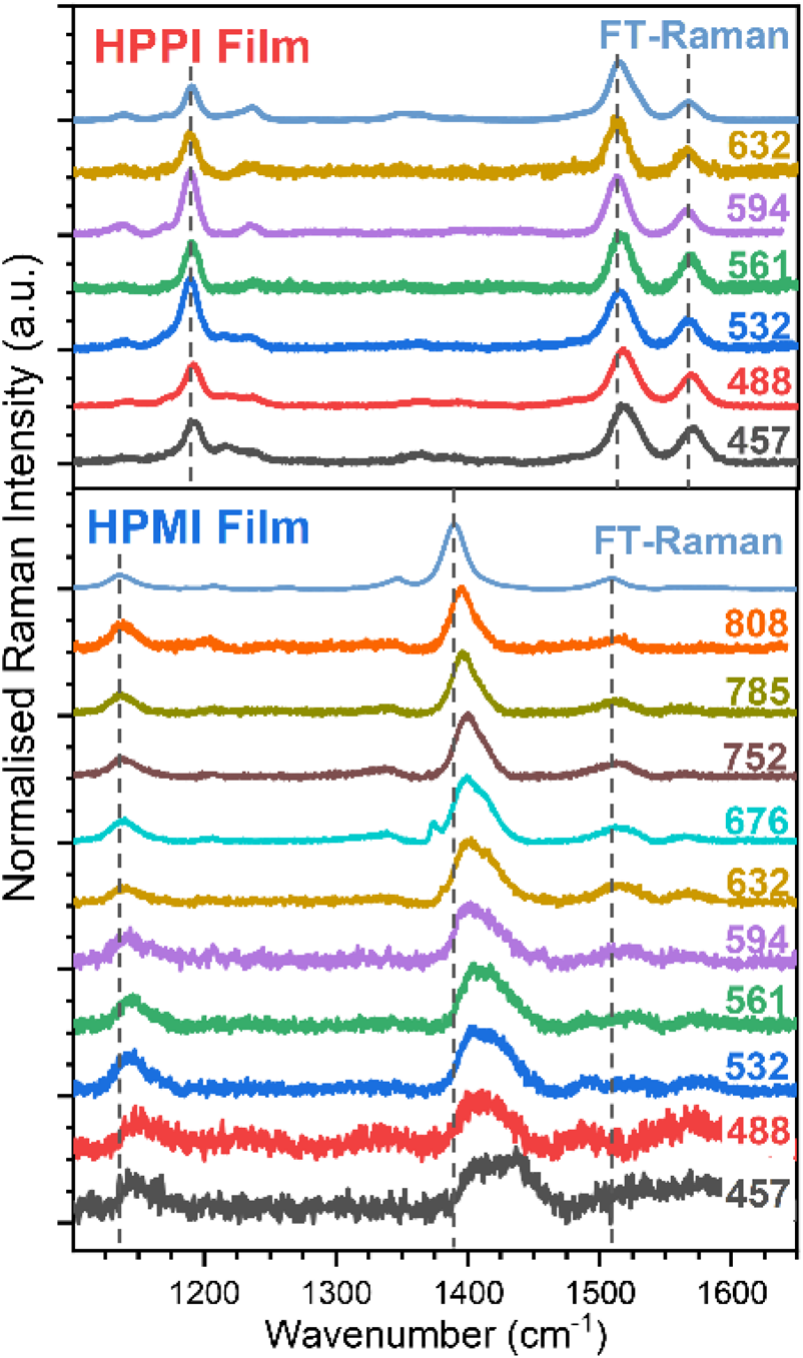
Resonance Raman spectra of HPPI (top) and HPMI (bottom) films exciting at different wavelengths. The power was maintained below 0.1 mW. Dashed lines have been added to help follow the band displacements.

To gain further insight into the electronic configuration of these materials, resonance Raman characterisation was performed (Fig. 4). The resonance Raman effect enhances the amplitude of those vibrational modes that mimic the structural changes occurring during the resonant electronic transition. Fig. 4 and S5† show the effect of altering the excitation wavelength on the HPPI polymer (normalised to the most intense band, 1528 cm^−1^). In general, there are no large differences in the spectra for HPPI, which confirms a very rigid and uniform HPPI polymer structure with a single resonant electronic transition. The most intense HPPI Raman band at 1528 cm^−1^, related to the C=C selenophene stretch, shows a slight (∼5 cm^−1^) downshift and narrowing of the peak when exciting at longer wavelengths. The downshift of a C=C mode is caused by a decrease in the effective bond force constant. A decreased force constant could be due to greater delocalisation of the π-electrons due to a more planar configuration.^33–37^ However, 5 cm^−1^ is close to the resolution of steady-state Raman spectroscopy, and thus indicates a uniform HPPI film with little conformer variability, as induced by the selenophene ring fusion. The appearance of the band at 1254 cm^−1^ when exciting at shorter wavelengths is discussed in the ESI.†

The changes in HPPI resonant Raman band intensity are summarised in excitation profiles (Fig. S6†). The bands at 1184 and 1272 cm^−1^, corresponding to the stretches of the bonds connecting HPPI monomers and the bond that fuses the selenophenes, respectively, maintain the same relative intensity as the HPPI main Raman band along the whole excitation wavelength range. The lack of variation in the excitation profile suggests these vibrational modes are not associated with the resonant electronic transition. However, the maleimide modes at 1232 and 1575 cm^−1^ exhibit a similar profile to one another, with enhancements in the relative intensity that mirror the electronic absorption. This indicates that the bonds associated with these vibrational modes also participate in the resonant electronic transition and thus suggests that the S_1_ excited state is largely localised over the phenyl group and the maleimide moiety.

The HPMI resonance Raman spectra (Fig. 4 and S7†) are considerably more complicated than those for HPPI. There are noticeable changes in the Raman spectrum with decreasing excitation wavelength, indicating a more inhomogeneous polymer structure (even in the solid phase), with the wide range of molecular environments being resonant in turn as the excitation wavelength is changed. The most intense non-resonant band at 1390 cm^−1^ progressively upshifts upon excitation at shorter wavelengths, indicating less π-electron delocalisation and, therefore, more amorphous domains or shorter chain segments – as could be induced by backbone twisting – are being excited at shorter wavelengths. The large 25 cm^−1^ upshift confirms HPMI's predicted greater backbone flexibility and wide range of different conformations, in contrast with the small 5 cm^−1^ upshift for the more rigid HPPI.

An interesting effect seen in the HPMI resonance Raman spectra was the rise of a new band at 1434 cm^−1^ upon excitation at shorter wavelengths. This new band can be explained by the assignment of the absorbance bands with the help of TD-DFT (Fig. S3†). According to TD-DFT calculations, the S_4_ state observed at 543 nm involves molecular orbitals more localised in the terminal units of the polymer. As the excitation wavelength approaches resonance with this new electronic state, the vibrational modes associated with these terminal units will be enhanced. This is consistent with both the increased contributions of terminal units as chain length decreases and the significant upshift of the main vibrational band when decreasing the length of the oligomer (Fig. S8†).

The results of Raman spectroscopy clearly confirm the rigidity and uniformity of the HPPI film morphology, with negligible variation of the Raman spectra with different excitation wavelengths. However, for HPMI, the large variation in Raman spectra with excitation density indicates a wide range of conformers in the film, suggesting substantially greater backbone flexibility. This provides the crucial experimental evidence that, as the calculations predicted, HPPI forms a more planar, rigid structure. As such, a reduced co-planarity cannot be the origin for HPPI's unusually blue-shifted absorbance.

Furthermore, it is important to note that the resonance Raman results in film are very similar to the ones obtained in solution phase (Fig. S4†), indicating that these significant differences in resonance Raman trends between HPMI and HPPI cannot be allocated to differing solid-state packing effects, only to the materials' intrinsic electronic structure. This also validates the *in vacuo* calculations as relevant to the experimental film data. Finally, it also demonstrates significant differences in the materials' electronic distribution, as seen by the large differences in the more intense vibrational modes for both polymers.

## Discussion

According to both experimental and calculated data, HPPI is clearly more rigid and planar than HPMI. Despite this, HPPI has an unusually high optical band-gap, leading to a strongly blue-shifted absorbance compared to its unfused analogue HPMI.

**Fig. 5:**
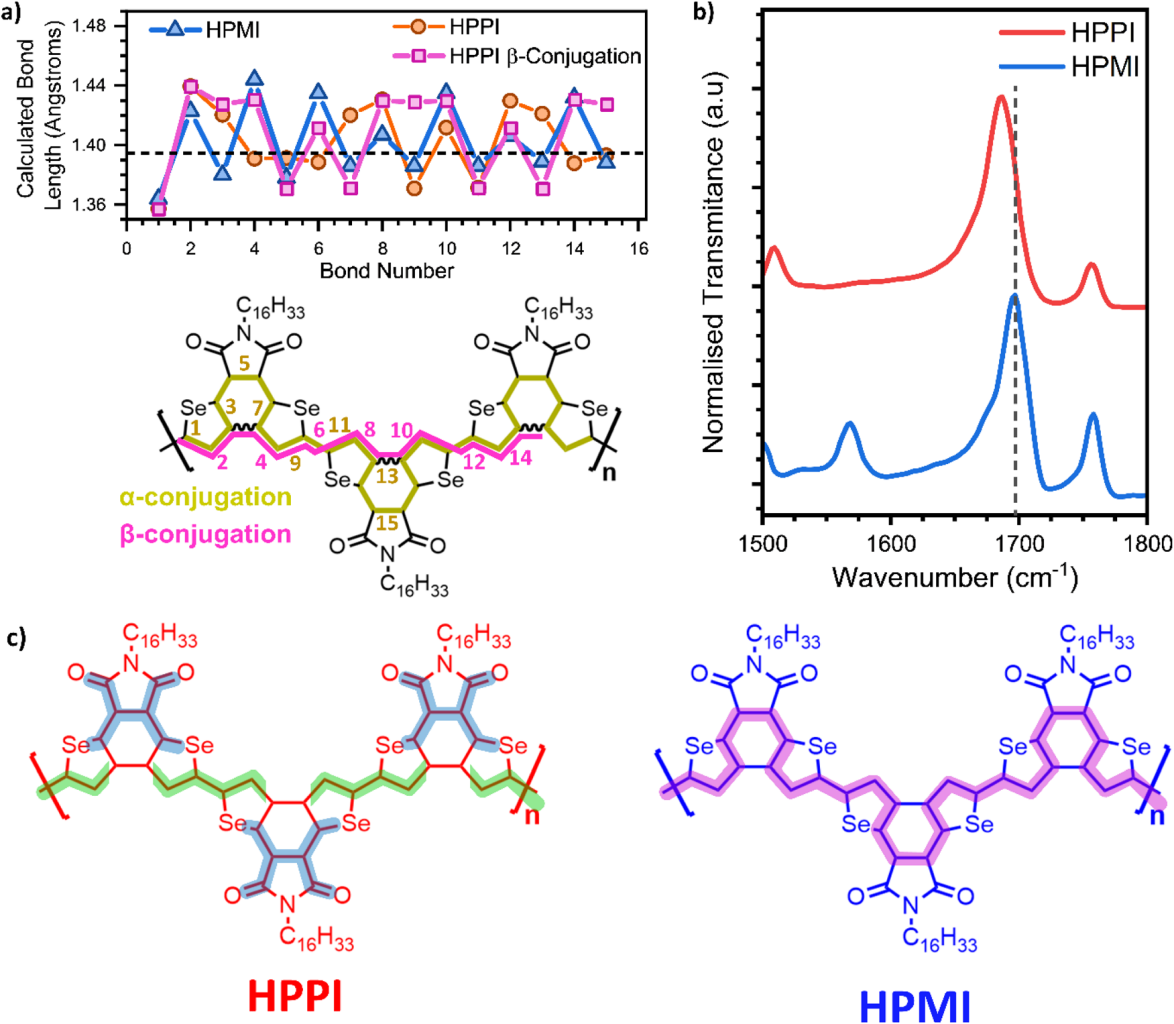
(a) Bond length diagram of HPMI (blue triangles) and HPPI through α (orange circles) and β-conjugation (pink squares) calculated with B3LYP/6-31G**. The dashed line represents the ideally aromatic C–C bond length taken from benzene.^38^ (b) Normalised IR spectrum of HPPI (blue) an HPMI (red) films. The dashed line has been added to remark the displacement of the C=O band. (c) Conjugation pathways for HPPI and HPMI. HPPI includes the truncated conjugation pathway (dark blue shadow) and the lack of β-conjugation (green shadow is hindered in the selenophene β-position).

To address the origin of HPPI's blue-shifted absorbance, we analyse conjugation pathways using bond length alternation (BLA) diagrams (Fig. 5a).^37,39–41^ HPMI shows a clear aromatic bond length alternation sequence. The bond lengths of the selenophene moiety (bonds 7, 8, and 9) are quite close to the aromatic optimum bond length (taken from benzene in vapour: 1.396 Å (ref. 38)), indicating a high degree of aromatisation of the central HPMI selenophene rings. In HPPI, however, no aromatic bond length alternation sequence exists along that same α-pathway. Indeed, two consecutive single bonds can be identified at bonds 2–3 and 7–8, creating a significant disruption to conjugation. The only region that matches the aromatic optimum is the region that joins the maleimide and the six-member ring (bonds 4, 5, and 6), but this is bookended by the consecutive single bonds, implying very short conjugation segments localised on the maleimide units. This is particularly interesting if we pay further attention to the asymmetry in the C–Se distance in the HPPI selenophene units (Fig. S9†): the C–Se bond linking with the maleimide moiety is shorter than the one that links with the other selenophene. This asymmetry in the C–Se bond length and thus distortion of the selenophene ring suggests a larger participation of the selenium atom in the conjugation, which is consistent with the calculated HOMO density distribution. The participation of the heteroatom with a higher degree of planarisation is in accordance with literature for polyheterocycles.^42^ This asymmetry in the C–Se bond together with the BLA seen in the maleimide moiety suggests the existence of a compressed, truncated conjugation pathway that goes from the selenium atom to the maleimide unit. This agrees with results obtained by IR spectroscopy (Fig. 5b), which shows the IR band associated with the carbonyl group for HPPI downshifted 12 cm^−1^ in comparison with the HPMI carbonyl band. The downshift indicates a decrease in double bond character, which is related to a higher contribution of the carbonyl to the conjugation pathway in HPPI.^43–46^ This truncated conjugation pathway is not electronically coupled with the backbone conjugation, as evidenced by the excitation profiles in the resonance Raman spectra, with the maleimide vibrational modes following different excitation profiles than selenophene modes.

HPPI conjugation through the bond that fuses the selenophenes must also be considered. However, the fusing bond is β-connected to the selenophene rings, and β-conjugation has been extensively shown in literature to be ineffective for thiophenes.^19,20^ This agrees with our observation that bonds 2–3–4, following this β-pathway (pink squares in Fig. 5a), all have appreciable single-bond character. This lack of efficient conjugation through selenophene β-connection hinders effective conjugation along the polymer backbone. As such, the main conjugation pathway possible in HPPI is within the maleimide units (Fig. 5c) and this is disrupted by the distorted selenophene rings. The conjugation length in HPPI is therefore short and this accounts for the larger-than-expected optical bandgap.

The disrupted conjugation predicted for HPPI is in agreement with its lack of selenophene aromaticity. The aromaticity was calculated using the HOMA (Harmonic Oscillator Model of Aromaticity) value for these materials (Fig. S9†). HOMA values are calculated using the length of each bond of a ring and comparing it with a “reference” (either single or double bond) number. HOMA values range from 0 to 1, where a value of 1 is purely aromatic (benzene-like). Despite its simplicity, it has been shown to give very good results for the calculation of aromatic character for organic polycycles.^47–50^ While the inner selenophenes of HPMI provide an expected HOMA value of 0.97, the disrupted HPPI selenophenes only give a HOMA value of 0.72, suggesting a considerably reduced aromaticity for HPPI.

**Fig. 6:**
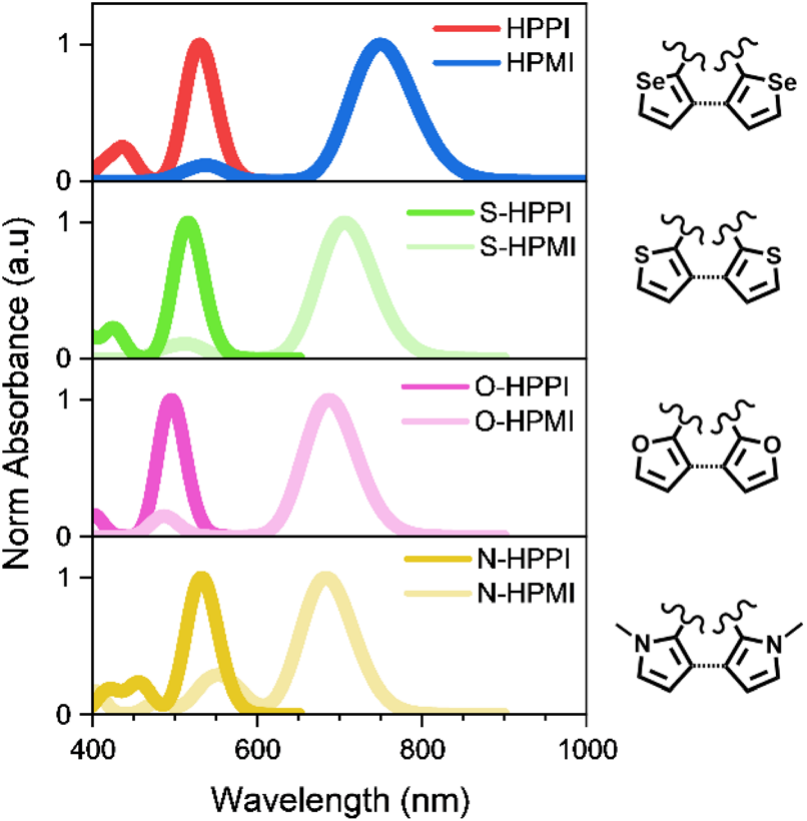
Calculated absorbance spectra of HPPI (red), HPMI (blue) and their derivatives with sulfur (green), oxygen (pink) and nitrogen (yellow) in their open (lighter colour) and closed ring (darker colour) forms. TD-DFT calculations were performed using a B3LYP/6-31G** level of theory.

To elucidate if this ‘truncated conjugation’ effect was specific to polycyclic polymers involving a heavy atom like selenium, we calculated the bond length, absorbance, and molecular orbitals of several HPPI and HPMI derivatives, substituting the selenium with other heteroatoms (S, O, and N). Fig. 6 and S10† show the results of these calculations. All the fused cycle analogues showed a blue-shifted absorbance in comparison with their open ring counterpart. As occurred in the selenophene materials, there was an asymmetry in the heterocycle C–X bond length (where X is the heteroatom). Moreover, there was a decrease in the 5-member ring aromaticity, as calculated by their HOMA value (Fig. S10†).

The origin for this unconventional difference in absorbance position can be explained in a similar fashion to the selenophene polymers. The increase of monomer planarity upon fusing the heteroatomic five-membered rings causes a higher contribution of the heteroatom to the conjugation and creates a truncated conjugation pathway from the heteroatom to the maleimide, completely uncoupled from the backbone conjugation. The truncated conjugation pathway diverts from the backbone through the β-connection of the five-member heteroatomic rings, which is considerably less efficient than in the α-position. The presence of this truncated conjugation mechanism in this variety of derivatives supports the generality of this counterintuitive mechanism and demonstrates its importance.

Crucially, this theoretical experiment also further confirms that the differences in HPPI and HPMI's electronic characteristics are not due to a difference in backbone planarity. The N-HPPI and N-HPMI pyrrole derivative structures are not planar due to the steric hindrance of the methyl groups attached to the nitrogen atoms (Fig. S11†): N-HPPI and N-HPMI have dihedral N–C–C–N angles of ∼38° and ∼42°, respectively. However, the fused analogue still maintains the same blue-shifted absorbance that we observe both theoretically and experimentally for HPMI and HPPI. This supports our hypothesis that a truncated conjugation pathway in HPPI is responsible for the blue-shifted absorbance, rather than a difference in polymer coplanarity.

To experimentally demonstrate the generality of the truncated conjugated mechanism, two analogous polymers, CPPI and CPMI, were analysed. Both CPPI and CPMI are copolymers containing a benzodithiazole moiety in the monomer backbone, but still possess the same fused and unfused selenophene ring structures as HPPI and HPMI.^18^ These two materials were fully characterised in similar fashion to HPPI and HPMI. As seen in Fig. S12,† the fused material, CPPI, had a larger bandgap than the unfused polymer, CPMI: an identical trend to HPPI and HPMI. The CPPI HOMO density was also shifted towards the β-connection of the selenophene ring, with a contribution from the Se atom itself. The resonance Raman spectra of CPPI and CPMI (Fig. S13†) also followed the same trends as HPPI and HPMI, with the fused CPPI displaying invariant Raman spectra with excitation wavelength while the unfused CPMI showed significant changes in Raman intensity profile with excitation wavelength. As with HPPI/HPMI, this strongly indicates a single invariant conformation for CPPI and considerably enhanced backbone flexibility and conformational variability for CPMI. Finally, the CPPI and HPPI BLA diagrams are very similar and showed the distinct lack of regular single/double bond alternation (Fig. S14†). This, together with the deviation of symmetry in the Se–C bond of the CPPI selenophenes and the CPPI planarity (in comparison with CPMI), confirms the existence of the truncated conjugation mechanism for CPPI as well. This represents additional evidence of the generality of the truncated conjugation mechanism.

To further understand the extension of the truncated conjugation mechanism, the influence of the maleimide moiety was studied through TD-DFT calculations on HPPI and HPMI derivatives in which the maleimide moiety is replaced with a vinylene group (Fig. S15†). As seen, for the derivatives without the maleimide, the fused selenophene material also shows a blue-shifted absorbance in comparison with the unfused one. This confirms that, despite the maleimide and its carbonyl groups being involved in the truncated conjugation pathway, they are not the main cause. Instead, the relationship of the vinylene group and its relative position respective to the heteroatom represents the pivotal aspect in the conjugation of these materials.^51,52^ This is of particular significance because it suggests that the truncated conjugation phenomenon could be extended to other fused ring blocks, not just maleimide. It also highlights the importance of the architecture of basic molecular units to understand materials for organic electronics. The relative position of the heteroatom in comparison with the 6-member ring is vital in this change in conjugation pathway.^53^

The manipulation of properties of closed/open heterocycles opens the door to the synthesis of a wide range of materials for multi-coloured photoswitches. This truncated conjugation provides several advantages over other organic photoswitches. It exhibits well separated absorption spectra for their open and closed forms, both materials absorbing in the visible spectrum (most photoswitches change from colourless to coloured).^54–58^ It also offers a blue-shift in the absorbance upon the photochemical reaction, when the conventional effect is an absorbance displacement to the IR upon photo-excitation.^59–63^

## Conclusions

We have studied two similar polymers based on conjugated selenophenes bonded through a maleimide moiety. HPPI had these selenophenes fused to induce a higher polymer planarisation, and therefore a larger effective conjugation was expected. However, the reverse was observed: fused HPPI exhibited a blue-shifted absorbance compared to the unfused HPMI, suggesting an unexpected decrease in HPPI's conjugation. Resonance Raman spectroscopy confirmed the rigidity of the HPPI polymer, where little spectral variation with changing excitation wavelength was observed, in comparison with large changes with the more flexible HPMI. Moreover, analysis of excitation profiles associated with the resonance effect showed a decoupling of HPPI's vibrational modes associated with the maleimide and selenophene moieties. The calculated bond lengths of the fused HPPI polymer suggests the monomer planarity distorts the selenophene rings and creates a truncated conjugation pathway localised primarily on the maleimide units, as verified using IR spectroscopy. This maleimide conjugated pathway is completely decoupled from the short conjugated segments along the polymer backbone, thereby largely decreasing the overall polymer backbone conjugation.

We demonstrate this to be a general phenomenon based on experimental data on analogous polymers, and calculations of derivative structures. The truncated conjugation is independent of the heteroatom used, and is only dependent on the relative position of the heteroatom. The elucidation of this truncated conjugation mechanism and its generality have major importance that goes beyond fundamental chemistry: this lack of conjugation was the origin of the poor mobility previously seen in HPPI. This work has established crucial structure-to-function relationships with important implication in the charge delocalisation and transport which are a pivotal point in the study of more efficient materials for organic electronics including transistors, solar cells, and molecular switches.

## Data availability

The results of the calculations will be included as Annex I in the ESI† as requested by the editor.

## Author contributions

J. M. M. B. performed and analysed the experiments, developed the research concept and prepared the first version of the manuscript. S. G. performed the theoretical calculations. H. G. performed additional experiments. T. M. C. developed the research concept, supervised the project and the manuscript writing. M. C. and M. A.-H. performed the materials synthesis and helped in the manuscript preparation. All authors have revised the manuscript.

## Conflicts of interest

There are no conflicts to declare.

## Supplementary Material

SC-014-D2SC06271B-s001
