# Supplementary material for: Truncated conjugation in fused heterocycle-based conducting polymers: when greater planarity does not enhance conjugation
Source: Chem Sci. 2022 Dec 7;14(4):812–21. doi: 10.1039/d2sc06271b (PMC9890783; doi:10.1039/d2sc06271b)
Supplement: SC-014-D2SC06271B-s001 [file SC-014-D2SC06271B-s001.pdf]

Electronic Supplementary Material (ESI) for Chemical Science.
This journal is © The Royal Society of Chemistry 2022

Electronic Supplementary Information

Truncated conjugation in fused heterocycle-based conducting polymers: when greater planarity does not enhance conjugation

Jose Manuel Marin-Beloqui,*[a,b] Sandra Gomez,[c] Hristo Gonev,[a] Marc Comí,[d] Mohammed Al-Hashimi,[d] Tracey M. Clarke.*[a]

[a] *Department of Chemistry*

University College London

Christopher Ingold Building, London WC1H 0AJ, United Kingdom

E-mail: tracey.clarke@ucl.ac.uk

[b] *Department of Physical Chemistry*

University of Malaga

Blvrd Louis Pasteur 31, 29010, Malaga, Spain

E-mail: jm.marinbeloqui@uma.es

[c] *Department of Physical Chemistry*

University of Salamanca

Caidos Sq., 37008, Salamanca, Spain

[d] *Department of Chemistry*

Texas A&M University at Qatar

Education City, Doha, P.O. Box 23874, Qatar

Experimental Section

Films were fabricated by drop-casting a 5 mg/mL chloroform solution of the HPMI and HPPI polymers. HPMI and HPPI were synthesized following the procedure described in our previous work.[1]

Ground state absorbance. Ground state absorbance was obtained with a Perkin Elmer Lambda 365.

Raman. Raman spectra were recorded using a custom-made back scattering setup with an Andor iDus 416 CCD camera attached to an Andor Shamrock 500i spectrograph. Raman signal was generated via excitation with a 6 ns, 10 Hz Nd:YAG laser (Spectra-Physics, INDI-40-10) for the excitation pulse. The intensity of the Raman excitation was decreased with the use of neutral density filters to maintain it at 0.1 mW, measured with an ES111C sensor (Thorlabs). The excitation wavelength was selected with a versaScan L-532 OPO and the appropriate notch filters were used in front of the spectrograph slits (200 mm).

FT-Raman data was obtained using a Bruker MultiRAM setup with an excitation wavelength of 1064 nm.

Infrared Spectroscopy. Data was obtained from a Bruker Alpha FT-IR Spectrometer.

Theoretical Section

The density functional theory (DFT) method was chosen to optimise the electronic ground state of the trimers HPMI and HPPI with the global hybrid B3LYP functional as well as long range corrected functionals such as CAM-B3LYP and ωB97X-D3. We performed optimisations with the 6-31G** and 6-311** basis sets. Frequencies were calculated at the B3LYP/6-31G** level of theory.

To decrease the calculation time and expense, the comparison between dimer, trimer and tetramer have been performed at the B3LYP/6-31G* level (Fig. S2).

Potential energy scans around the Se-C-C-Se dihedral bond that connects two monomeric units were carried out using B3LYP/6-31G** in both HPMI and HPPI trimers. For each manually fixed Se-C-C-Se torsion angle (from -180 to 180 degrees) of one of the terminal monomeric units, a geometry optimisation has been performed at the B3LYP/6-31G** level of theory, letting the other degrees of freedom reach their minimal energy conformation.

Vertical energies at the optimised geometries were computed with linear response time dependent density functional theory (LR-TDDFT) using the B3LYP and the 6-31G** and 6-311** basis sets.

The simulations were carried out with QChem5.4.

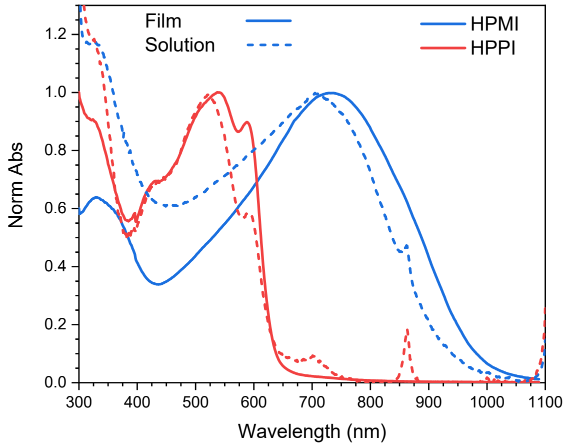

Fig. S1. UV-Vis absorption spectra of 10^{-4} M chloroform solution vs thin film for homopolymers HPMI (blue) and HPPI (red).

Table S1. Molecular mass and polydispersity of HPPI and HPMI polymers.

	Mn (kDa)	PDI
**HPPI**	4.4	1.9
**HPMI**	6.1	1.4

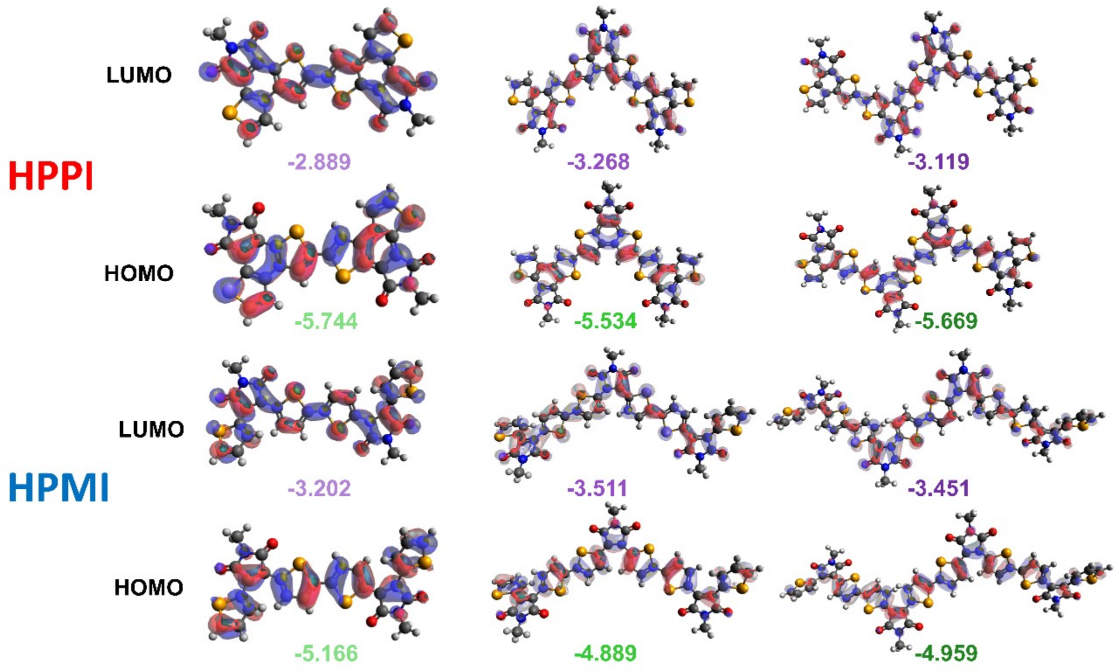

Fig. S2. Calculated orbitals for HOMO and LUMO levels for HPPI (top) and HPMI (bottom) dimer, trimer and tetramers using B3LYP/6-31G* level TD-DFT calculations. Energy LUMO (purple) and HOMO (green) levels for those molecules are also displayed.

Table S2. Se-C-C-Se torsional angles in degree from trimer optimised structures calculated with different functionals and basis sets. TZ corresponds to the triple zeta Pople basis set 6-311G** and DZ to 6-31G**. CAM is an abbreviation for CAM-B3LYP. There are two torsional angles per trimer, corresponding to the two Se-C-C-Se torsional angles that link the three maleimide monomer units.

	Dihedral Angle (°)			
	HPPI		HPMI	
**B3LYP-DZ**	0.24	0.07	15.85	15.11
**B3LYP-TZ**	3.57	2.63	15.57	15.18
**CAM-DZ**	0.07	0.00	20.39	20.27
**CAM-TZ**	0.35	0.06	31.70	31.40
**ωB97X-D3-DZ**	0.32	0.29	25.59	25.41

a)

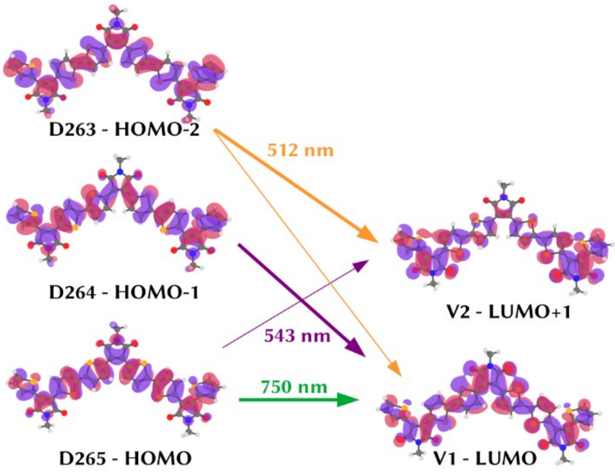

c)

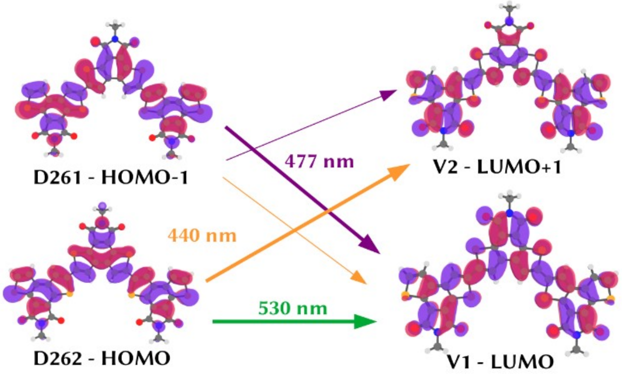

b)

Excited state 1:
Excitation energy (eV) = 1.6538
Strength: 2.318235665
D(265) --> V(1) amplitude = 0.9809

Excited state 4:
Excitation energy (eV) = 2.2847
Strength: 0.2376763151
D(264) --> V(1) amplitude = 0.7520
D(265) --> V(2) amplitude = 0.5930

Excited state 5:
Excitation energy (eV) = 2.4241
Strength: 0.1144764238
D(263) --> V(1) amplitude = -0.5562
D(264) --> V(2) amplitude = 0.8103

d)

Excited state 1:
Excitation energy (eV) = 2.3402
Strength: 1.3420303024
D(262) --> V(1) amplitude = 0.9816

Excited state 2:
Excitation energy (eV) = 2.6005
Strength: 0.0707600731
D(261) --> V(1) amplitude = 0.8834
D(262) --> V(2) amplitude = -0.4292

Excited state 3:
Excitation energy (eV) = 2.8179
Strength: 0.2695802308
D(261) --> V(1) amplitude = 0.3661
D(262) --> V(2) amplitude = 0.8305

Fig S3. Frontier molecular orbitals involved in the main transitions of **a)** HPMI and **c)** HPPI calculated with TD-B3LYP/6-31G**. D stands for "doubly occupied" whereas V are virtual orbitals. Extract from output specifying contributions to the brightest excited states of **b)** HPMI and **d)** HPPI calculated with TD-B3LYP/6-31G**.

Table S3. Absorption energies of the main peak in eV and in nm (in parentheses). The vertical energies have been calculated at the geometries optimised at the same level of theory.

	Calculated vertical energies (eV/nm)			
	HPPI		HPMI	
	DZ	TZ	DZ	TZ
**B3LYP**	2.34 eV (529.78 nm)	2.32 eV (534.53 nm)	1.65 eV (749.67 nm)	1.64 eV (756.16 nm)
**ωB97X-D3**	3.21 eV (385.71 nm)	3.18 eV (389.29 nm)	2.57 eV (483.11 nm)	2.54 eV (487.46 nm)

Raman Characterisation

The most intense HPPI experimental Raman band (1528 cm^{-1}) corresponds to the calculated vibrational modes at 1512 and 1519 cm^{-1}. These calculated bands correspond to the C=C stretch along the selenophene backbone. Furthermore, the calculated 1519 cm^{-1} mode also shows a strong contribution from the central fused benzene ring CC stretching corresponding to the bond linking the selenophene and the maleimide, while the 1512 cm^{-1} mode contains terminal benzene stretches. The latter is less likely in a long-chain polymer, and thus we expect the experimental band to be dominated by the central benzene stretch combined with the selenophene C=C stretch. The bands in the range from 1200 to the 1300 cm^{-1} belong to vibrational modes associated with the benzene ring in the HPPI. Particularly, the experimental band at 1272 cm^{-1} was assigned to the bond that fuses the selenophenes (C3-C11), which is obviously not present in the HPMI polymer. The experimental band at 1575 cm^{-1} was assigned to the calculated band seen at 1543 cm^{-1}, which related to CC stretching of the benzene bonds that connect the maleimide moiety with the selenophenes. This vibrational mode was also contributing to the most intense HPPI band.

HPMI showed a very different Raman spectrum due to the absence of the fused-ring CC bond (C3-C11), thereby negating benzene-related vibrational contributions to the modes. HPMI shows its most intense band at 1390 cm^{-1}, associated with the calculated selenophene C-C stretch at 1389 cm^{-1}. The band experimentally seen experimentally at 1207 cm^{-1}, corresponding to the calculated vibrational mode at 1183 cm^{-1}, was associated to the C-C stretch of the bond that connect HPMI monomers (C13-C14). This mode has similar position and intensity to HPPI (experimentally seen at 1184 cm^{-1}).

A remarkable difference between HPPI and HPMI Raman spectra are the bands in the 1500-1600 cm^{-1} region. The HPPI has high and medium intensity bands that were associated with the C=C stretch of the selenophene conjugation and the bonds connecting the maleimide and selenophene (C8-C9 and C5-C6), respectively. The HPMI band seen experimentally in this region at 1509 cm^{-1} was associated with the stretch of the maleimide double bond (C5-C9 and C18-C18b). The band associated with this same vibration in the HPPI appears in the calculated spectrum at 1468 cm^{-1}, largely displaced to lower frequencies with negligible intensity. This large difference in both intensity and displacement means that fusing the selenophenes has a huge impact in the maleimide vibrational force constant.

Another difference with the HPPI polymer is the band seen at 1348 cm^{-1} in the HPMI experimental Raman spectrum, associated with the selenophene breathing. There was no equivalent vibrational mode for HPPI in the spectrum for the calculated range. This lack of vibrational breathing mode in the HPPI suggests a lack of aromaticity on the HPPI selenophene ring in comparison with the HPMI.

In addition, a new band rises at 1254 cm^{-1} associated with the maleimide stretches of the terminal monomers when exciting at shorter wavelengths. At shorter wavelengths the shorter length polymers are going to be excited, and therefore this vibrational mode has higher intensity because the terminal monomers are going to represent a larger portion of the smaller chain polymers in comparison to their proportion in longer chain polymers.

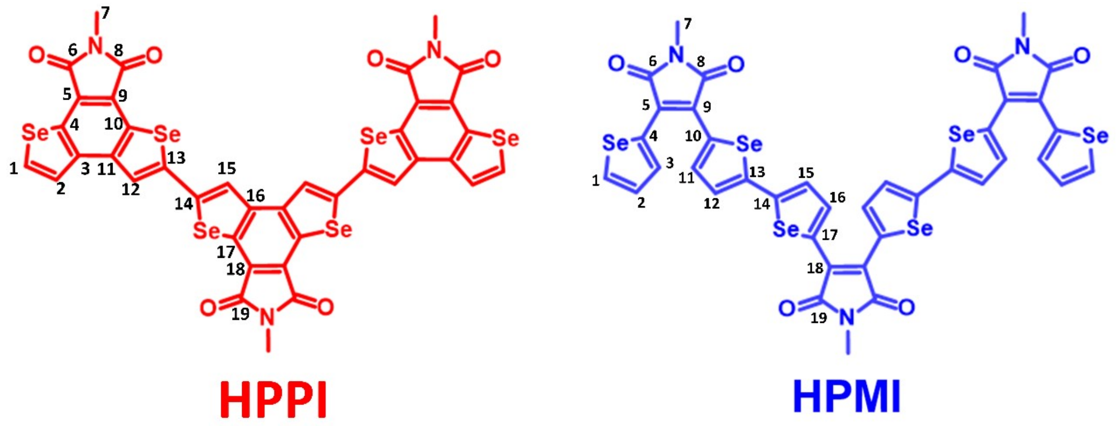

Scheme S1. Atom labels used for the HPPI and HPMI Raman band assignment.

Table S4. Table summarising the main vibrational modes for HPPI and HPMI Raman spectra and their correspondence with the calculations. The intensity of each band was indicated between parenthesis next to the frequency: (s) strong, (m) medium and (w) weak.

	**HPPI**			**HPMI**		
**Mode Description**	**Mode Number**	**Calc υ (cm^{-1})**	**Exp υ (cm^{-1})**	**Mode Number**	**Calc υ (cm^{-1})**	**Exp υ (cm^{-1})**
C_3-C_{11} (Selenophene fusing bond)	**148**	1249 (w)	1272 (m)			
Maleimide-Selenophene (C_4-C_5/C_9-C_{10}/C_{17}-C_{18}) Stretch	**180**	1543 (w)	1575 (m)			
Maleimide C-C Stretch (C_5-C_6/C_8-C_9/C_{18}-C_{19})	**141**	1213 (m)	1232 (m)	**147**	1124 (m)	1137 (m)
Maleimide C=C stretch (C_5-C_9)				**190**	1496 (m)	1509 (m)
Selenophene C=C Conjug. stretch	**177/ 178**	1512/ 1519 (s)	1528 (s)	**173**	1389 (s)	1390 (s)
Selenophene Breathing				**166/ 168**	1320/ 1355 (m)	1348 (m)
Sephene-Sephene (C_{13}-C_{14}) Stretch	**138**	1167 (w)	1184 (w)	**153**	1183 (w)	1207 (w)

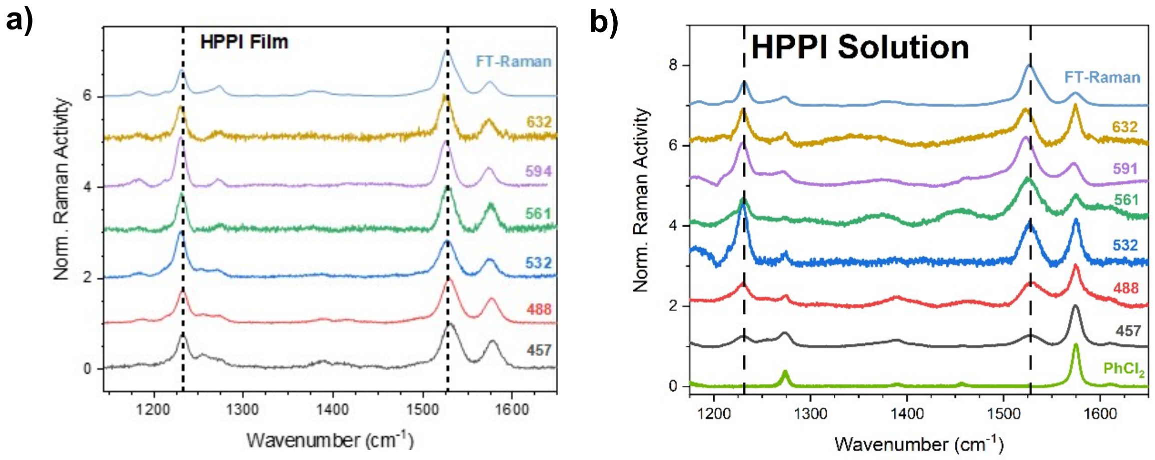

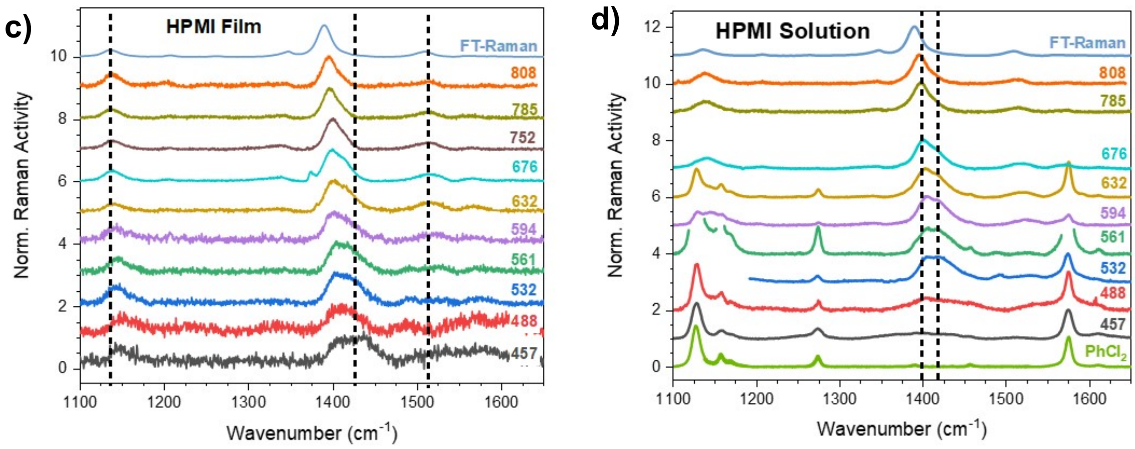

Fig. S4. Comparison of the resonance Raman effect in film (a and c) and solution (b and d) for HPPI (a and b) and HPMI (c and d). Dichlorobenzene has been added to recognise the bands associated with the solvent.

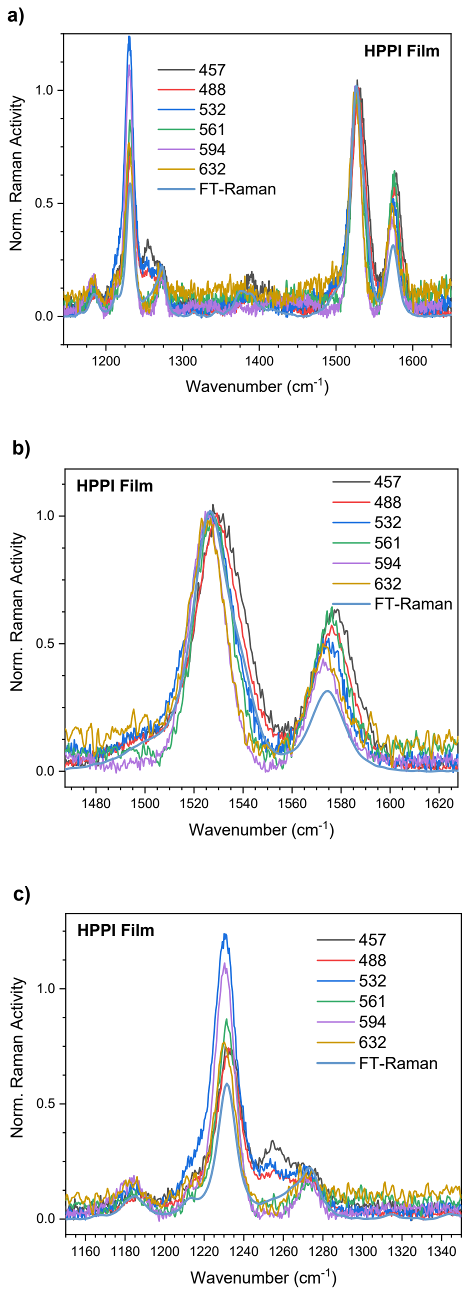

Fig. S5. Normalised to 1 Resonant Raman spectra for HPPI film. The power was maintained below 0.1 mW.

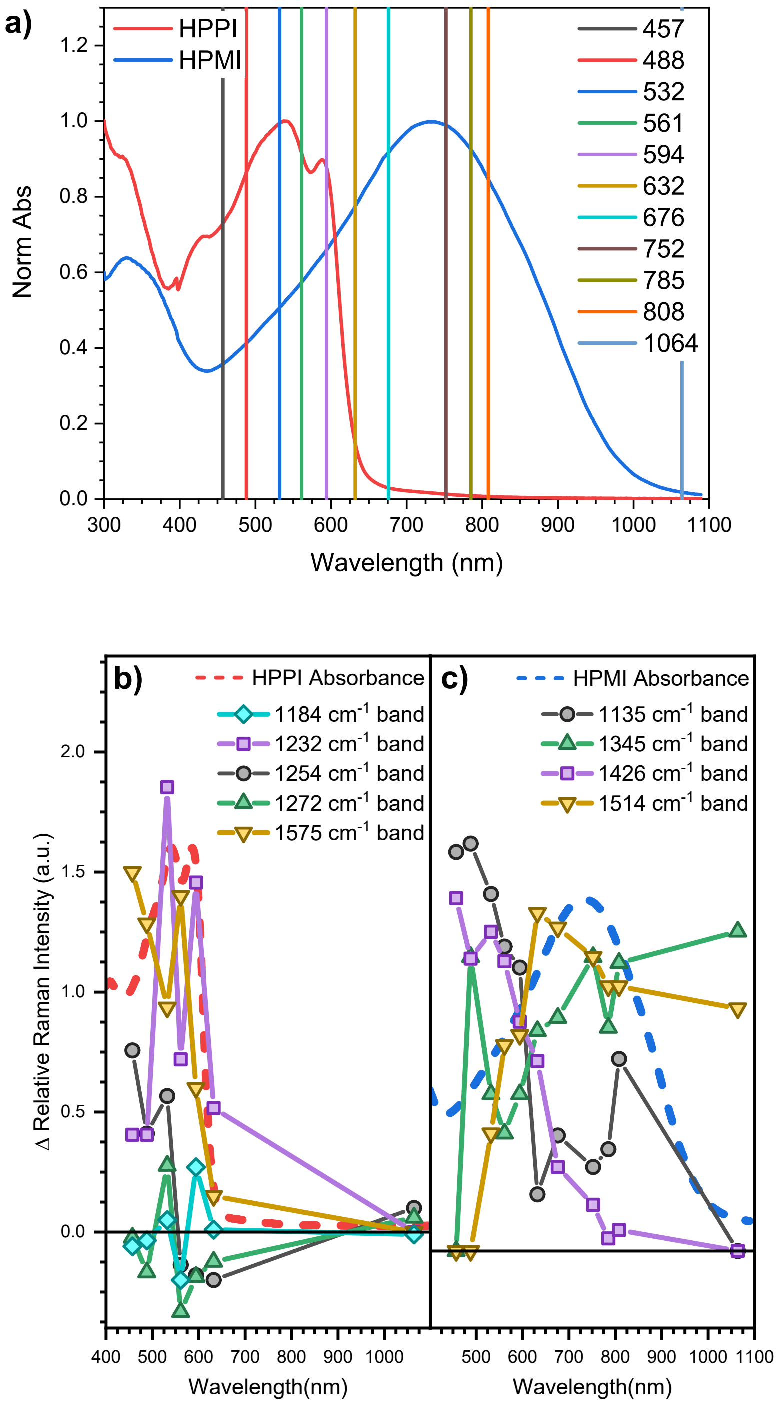

Fig. S6. **a)** Normalised absorption spectra for HPPI (red) and HPMI (blue) films and the position of the different excitation wavelengths used for the resonance Raman experiment. Normalised excitation profile of the Raman bands for **b)** HPPI and **c)** HPMI. The excitation profile was obtained normalising the corresponding band to the most intense band (1528 and 1390 cm^{-1} for HPPI and HPMI, respectively).

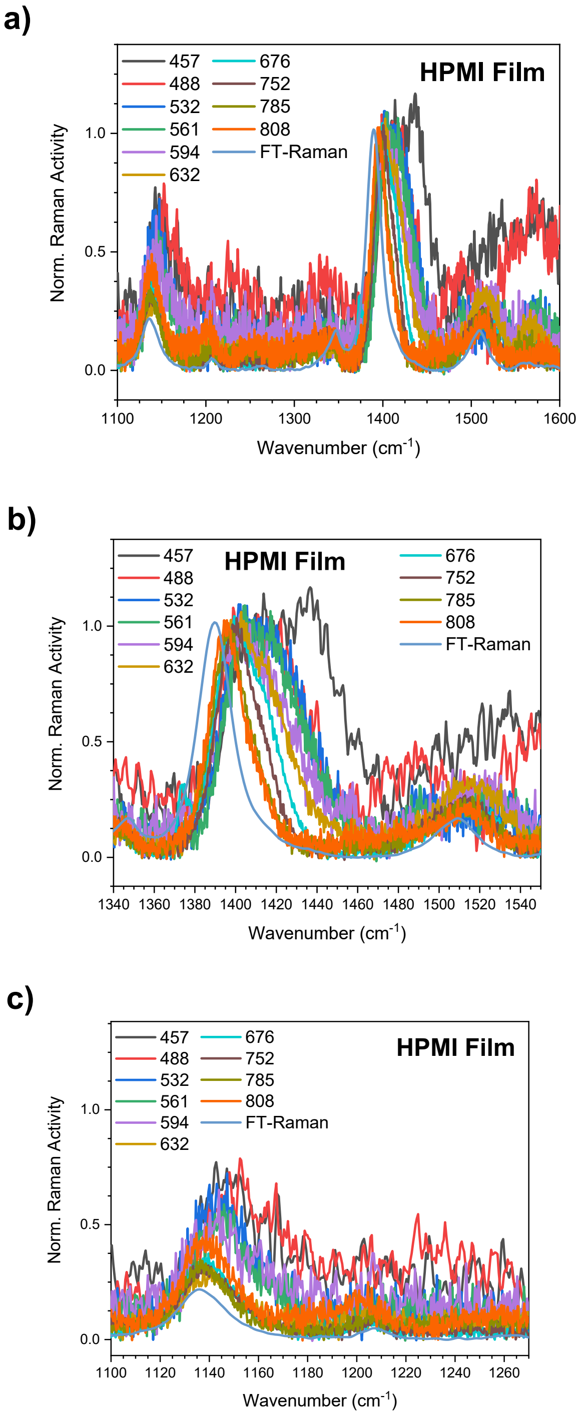

Fig. S7. Normalised to 1 Resonant Raman spectra for HPMI film. The power was maintained below 0.1 mW.

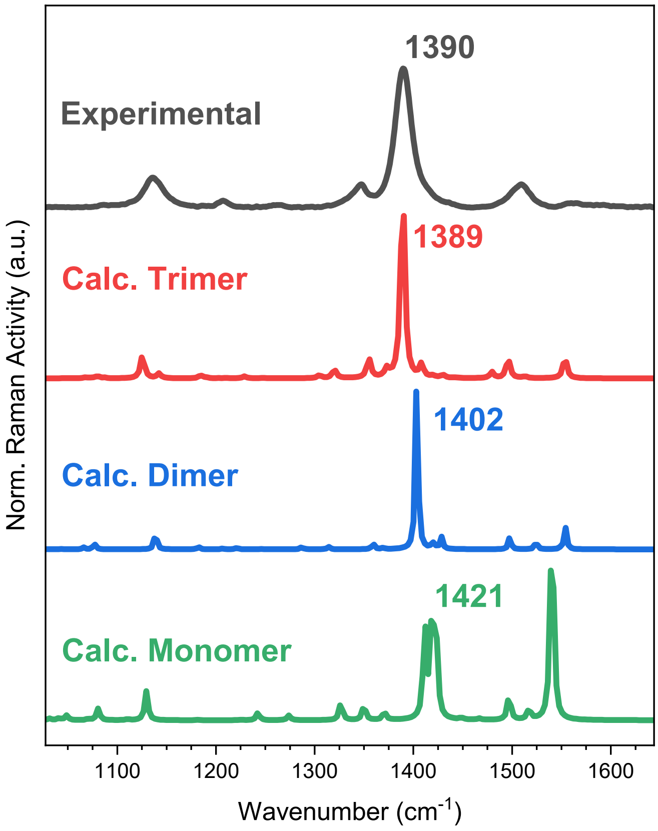

Fig. S8. Comparison of the calculated Raman spectra an HPMI trimer (red), dimer (blue) and monomer (green). The experimental FT-Raman of an HPMI film was added as comparison. Calculations were carried out at B3LYP/6-31g** level.

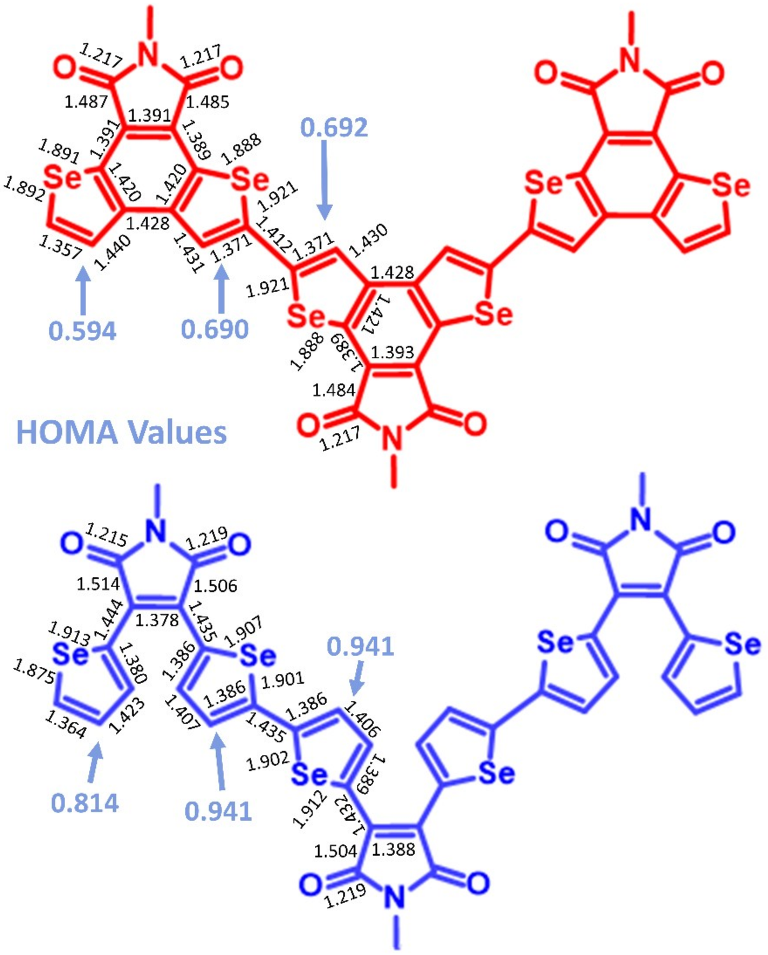

Fig. S9. Calculated bond length for HPPI and HPMI. HOMA values for selenophenes are in light blue. Calculations were performed at B3LYP/6-31G** theory level.

Calculation of the HOMA value

To calculate the HOMA value the following formula was used:[2]

$$HOMA = 1 - \frac{\alpha}{n}\sum\left(R_{opt} - R_{ij}\right)^2 \quad \textbf{(1)}$$

where n was the number of members of the ring and R_{ij} the calculated bond length for each of the bonds of the ring. α and R_{opt} were obtained from literature,[2] where the authors calculated them using the following formulae:

$$R_{opt} = \frac{\left(k_{(s)}R_{(s)} + k_{(d)}R_{(d)}\right)}{k_{(s)} + k_{(d)}} \quad \textbf{(2)}$$

$$\alpha = \frac{2}{\left[\left(R_{(s)} + R_{opt}\right)^2 + \left(R_{(d)} + R_{opt}\right)^2\right]} \quad \textbf{(3)}$$

Where k is the force constant for the bonds, 2 and 1 for $k_{(d)}$ and $k_{(s)}$, respectively. $R_{(s)}$ and $R_{(d)}$ are the reference single and double bonds.

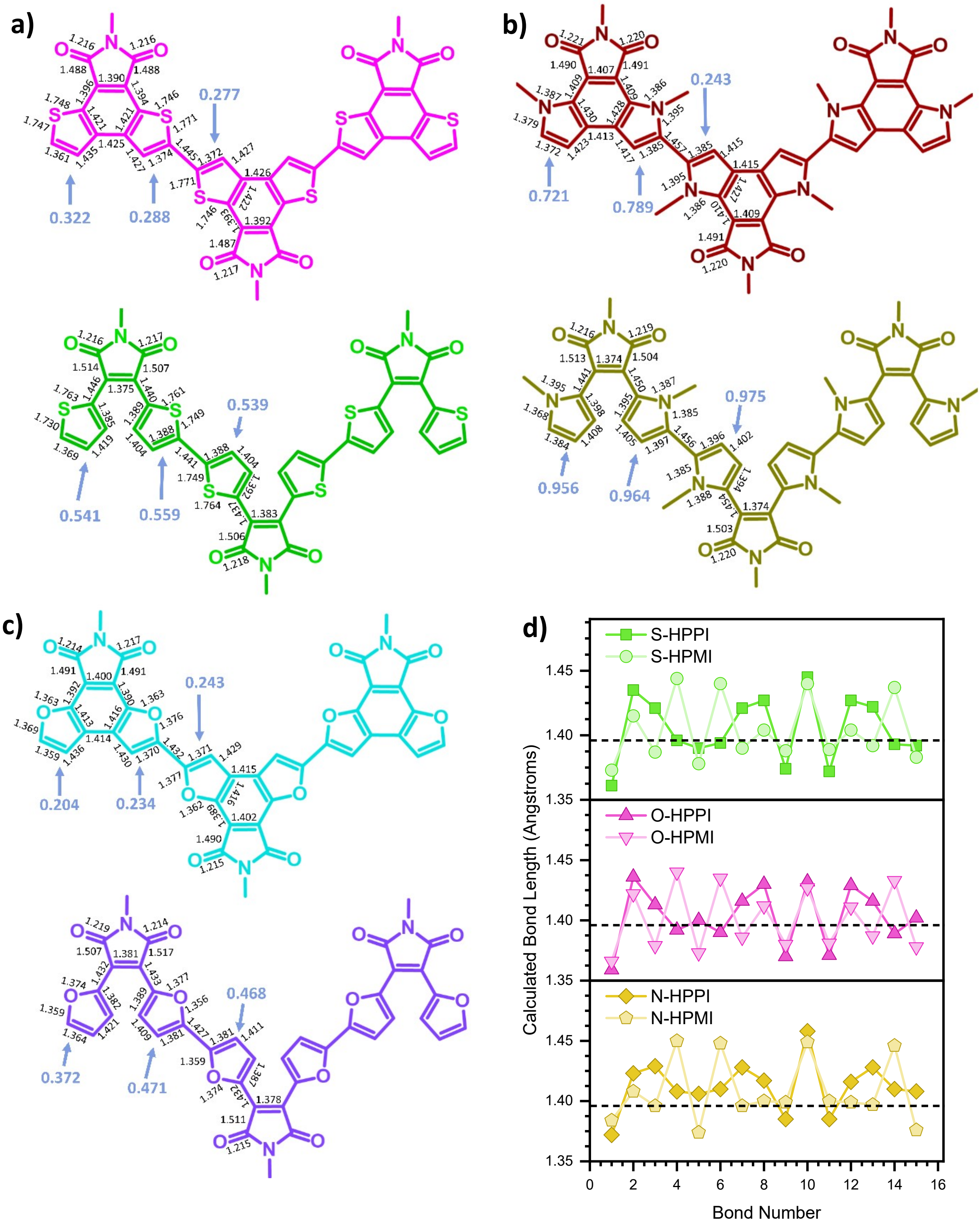

Fig. S10. Calculated bond lengths for HPPI and HPMI derivatives exchanging selenium with **a)** sulfur, **b)** oxygen and **c)** nitrogen. **d)** BLA diagram with the bond lengths calculated for the HPPI and HPMI derivatives. Calculations were performed at B3LYP/6-31G** theory level.

a)

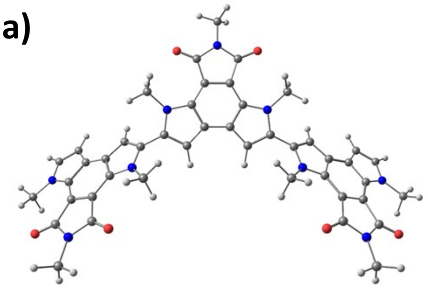

b)

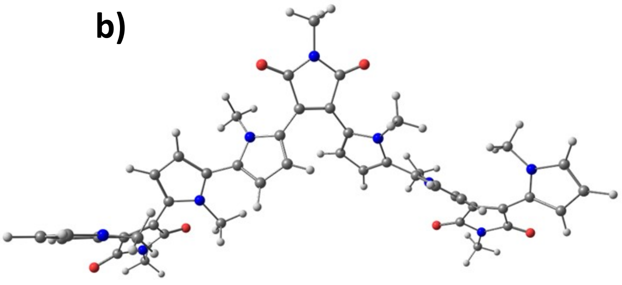

c)

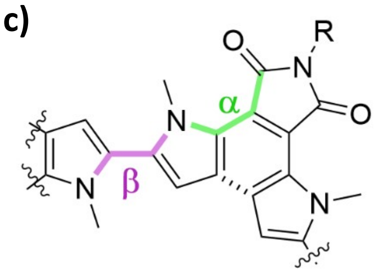

	N-HPPI		N-HPMI	
**Intra-monomer Dihedral angle (α)**	5.23°	5.28°	51.43°	41.99°
**Inter-monomer Dihedral angle (β)**	38.46°	38.82°	42.71°	41.07°

Fig. S11. Ground state optimised geometry calculated at the levels of theory B3LYP/6-31G**of **a)** N-HPPI trimer and **b)** N-HPMI trimer. c) Dihedral angles of N-HPPI and N-HPMI calculated at the level B3LYP/6-31G**.

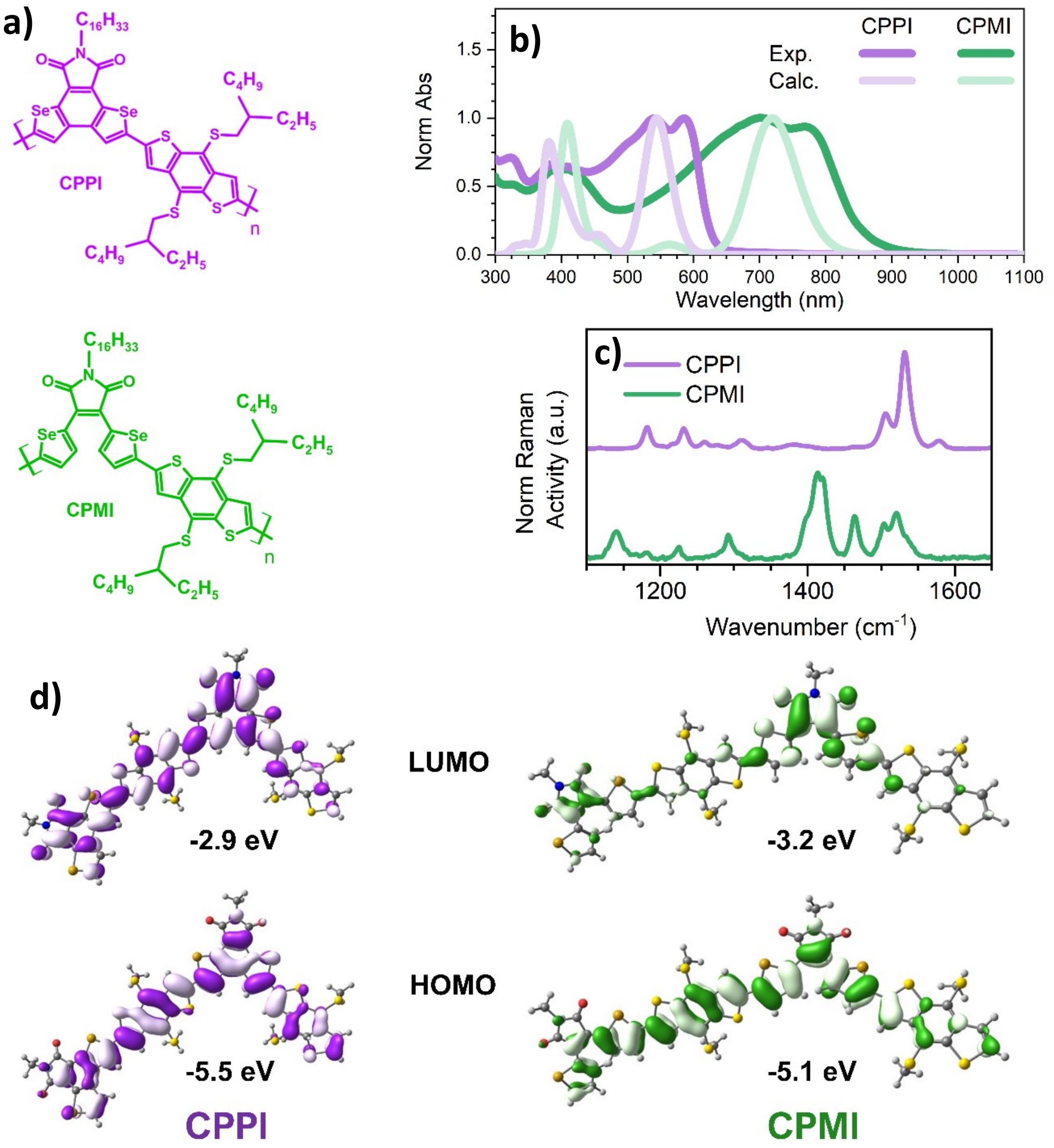

Fig. S12. a) Molecular structures of CPPI (purple) and CPMI (green) polymers. **b)** Normalised ground state absorbance of CPMI and CPPI films along with their calculated absorbance. **c)** FT-Raman spectra of CPPI and CPMI films. **d)** Calculated HOMO (bottom) and LUMO (top) molecular orbitals of CPPI and CPMI dimers and their corresponding calculated energies. All calculations have been performed with a theory level of B3LYP/6-31G**.

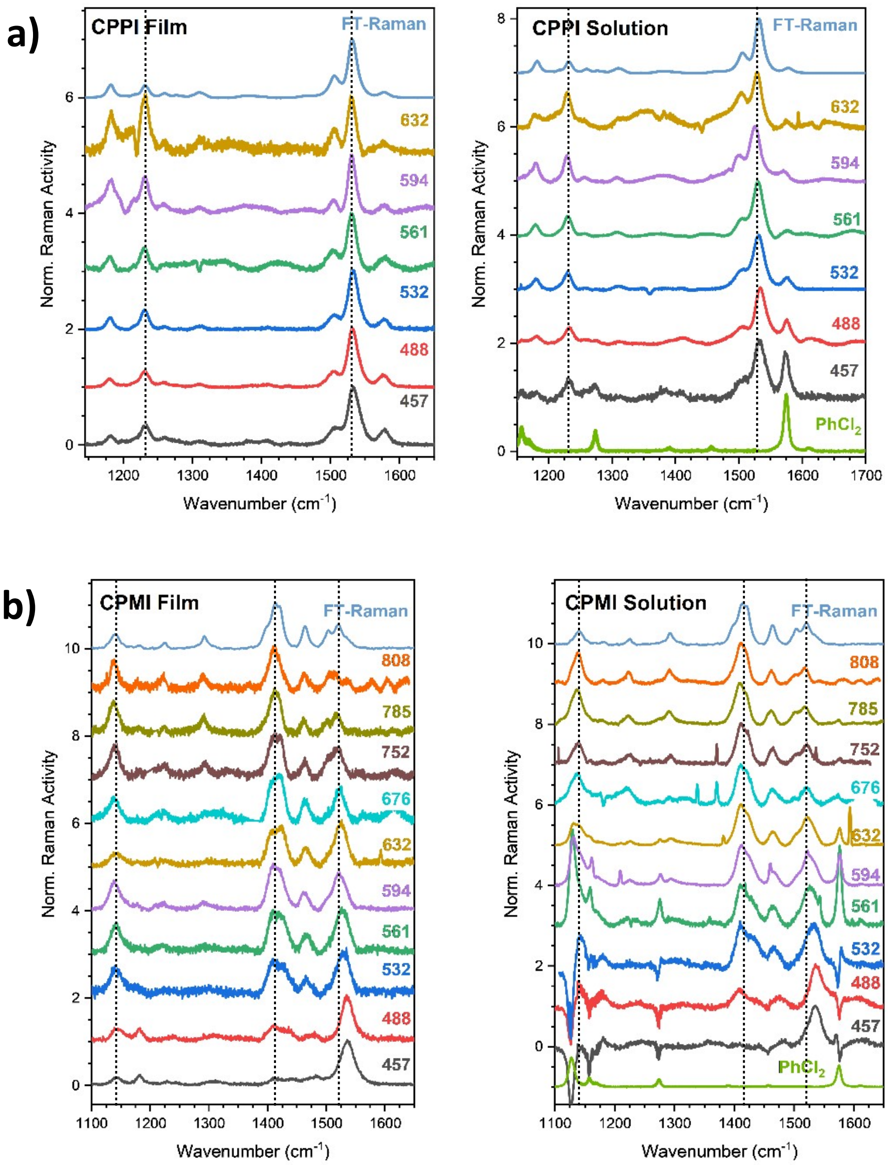

Fig. S13. Comparison of the resonance Raman spectra in solutions and film of **a)** CPPI and **b)** CPMI.

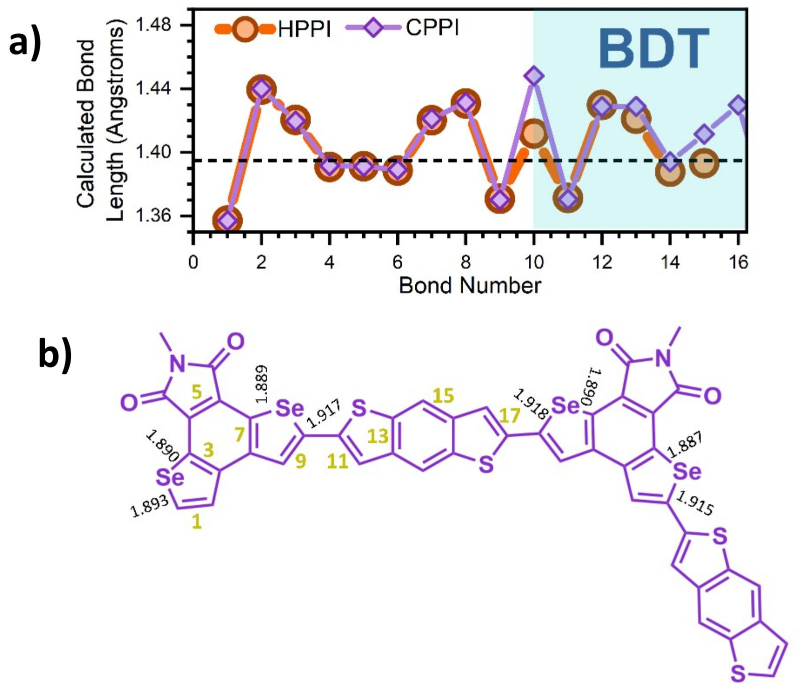

c)

	IntraMon Angle (°)	InterMon Angle (°)
CPPI	0.02	0.87
CPMI	26.64	0.59

Fig. S14. a) Bond length alternation diagram comparing the values for HPPI and CPPI. The blue transparent square represents the values corresponding the BDT unit in CPPI. **b)** C-Se bond length of the 5-member rings of CPPI as well as the atom labels (in gold) used for **a)** CPPI BLA. **c)** Calculated dihedral angles inside the monomer and of the intermonomeral bond. All calculations have been performed with a theory level of B3LYP/6-31G**.

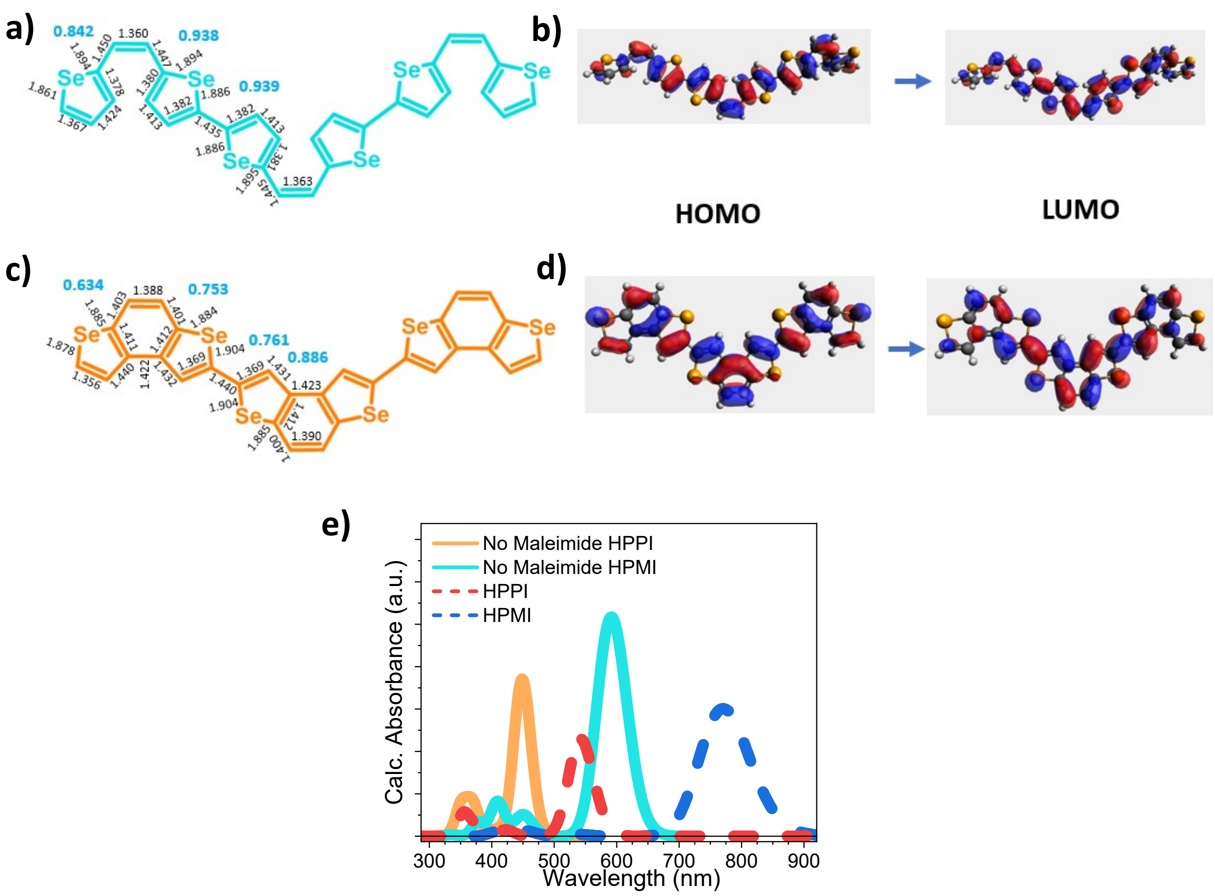

Figure S15. Results of the calculations for the HPPI and HPMI derivatives without the maleimide moiety. Bond length of the derivative without maleimide of a) HPMI and c) HPPI as well as the calculated HOMO and LUMO orbitals responsible for the main electronic transition. The calculated absorbance for both materials is shown in e), where the dashed lines represent the calculated HPPI (red) and HPMI (blue) absorbance. Calculations were performed at B3LYP/6-31G* level.

Bibliography

(1) Comí, M.; Ocheje, M. U.; Attar, S.; Mu, A. U.; Philips, B. K.; Kalin, A. J.; Kakosimos, K. E.; Fang, L.; Rondeau-Gagné, S.; Al-Hashimi, M. Synthesis and Photocyclization of Conjugated Diselenophene Pyrrole-2,5-Dione Based Monomers for Optoelectronics. *Macromolecules* **2021**, *54* (2), 665–672. https://doi.org/10.1021/acs.macromol.0c02021.

(2) Krygowski, T. M. Crystallographic Studies of Inter- and Intramolecular Interactions Reflected in Aromatic Character of .Pi.-Electron Systems. *J Chem Inf Comput Sci* **1993**, *33* (1), 70–78. https://doi.org/10.1021/ci00011a011.

Annex I – Calculations Cartesian Coordinates

HPPI (B3LYP-DZ)

1	C	6.1788830649	-2.8077037009	0.0025962719
2	C	5.4658512408	-1.6304647913	0.0022595581
3	C	7.5584807620	-2.8013267070	0.0020546654
4	C	6.1536570066	-0.4095015138	0.0006021591
5	C	5.2842582828	0.7463994716	-0.0003508013
6	H	5.6805090199	1.7415187955	-0.0017443081
7	C	3.9617750468	0.5074961815	0.0006286248
8	C	8.4269763798	0.7664170059	-0.0016418061
9	H	8.0415133361	1.7652677926	-0.0028172993
10	C	7.5626423820	-0.4011544394	0.0000410695
11	C	8.2629251059	-1.6175543903	0.0011576620
12	C	8.0212595870	-4.2094506015	0.0042201270
13	C	5.7239907213	-4.2164380481	0.0052041150
14	N	6.8743423978	-4.9908595450	0.0092913461
15	C	6.8793597264	-6.4443380653	-0.0036548593
16	H	5.9872754410	-6.7976807648	0.4879704341
17	H	6.9008694362	-6.8227686597	-1.0163866719
18	H	7.7524440430	-6.7939036307	0.5236657059
19	O	9.1623197519	-4.6234719830	0.0045012540
20	O	4.5835094123	-4.6335563262	0.0065090857
21	Se	10.1578550290	-1.3570696586	0.0008447638
22	Se	3.5723247880	-1.3908392379	0.0032185369
23	C	9.7380763019	0.5099404370	-0.0020449955
24	H	10.5244463702	1.2327381676	-0.0033180432
25	C	2.8932562315	1.4847945631	0.0001318109
26	C	1.5696714643	1.2505156890	0.0000617279
27	Se	3.2892176209	3.3818750206	-0.0005209570
28	H	1.1719258373	0.2560518762	0.0003202833
29	C	0.7047484567	2.4096075348	-0.0007467567
30	C	1.3973852642	3.6293847596	-0.0011254581
31	C	-0.7047517022	2.4096073084	-0.0012887414
32	C	0.6902565061	4.8091462249	-0.0012986168
33	C	-1.3973888069	3.6293838322	-0.0021529600
34	C	-1.5696739118	1.2505136940	-0.0010564163
35	C	-0.6902573563	4.8091468612	-0.0017827161
36	C	1.1487049132	6.2171584611	-0.0026676685
37	Se	-3.2892196236	3.3818718002	-0.0032097302
38	H	-1.1719270768	0.2560505888	-0.0001658449
39	C	-2.8932579334	1.4847914676	-0.0017264198
40	C	-1.1487605257	6.2171438827	-0.0033965890
41	N	-0.0000180832	6.9949643642	-0.0071578939
42	O	2.2898449631	6.6321928195	-0.0028206053
43	O	-2.2899557215	6.6320343116	-0.0044833474
44	C	0.0001931399	8.4486625181	0.0065539529
45	H	-0.8862386026	8.7999921151	-0.4966064480
46	H	0.0074102164	8.8264658904	1.0196818679
47	H	0.8791238127	8.8005953713	-0.5092638830
48	C	-3.9617771896	0.5074932062	-0.0015939103

49	C	-5.2842560526	0.7463941880	0.0022770355
50	Se	-3.5723342241	-1.3908363499	-0.0071905630
51	H	-5.6804995416	1.7415106284	0.0059067209
52	C	-6.1536566173	-0.4095051934	0.0017167450
53	C	-5.4658575467	-1.6304644338	-0.0030731051
54	C	-7.5626367613	-0.4011616735	0.0055268414
55	C	-6.1788898799	-2.8076987947	-0.0047398543
56	C	-8.2629213341	-1.6175603052	0.0044728184
57	C	-8.4269628150	0.7664064732	0.0106797310
58	C	-7.5584825345	-2.8013265676	-0.0010304532
59	C	-5.7240566625	-4.2164468657	-0.0085041779
60	Se	-10.1578402784	-1.3570813908	0.0108632322
61	H	-8.0414959074	1.7652557713	0.0119025757
62	C	-9.7380592067	0.5099297739	0.0134476891
63	C	-8.0213178505	-4.2094355849	-0.0025489804
64	N	-6.8743787343	-4.9908688611	-0.0037180405
65	O	-4.5836350335	-4.6337040486	-0.0123620590
66	H	-10.5244238776	1.2327246141	0.0172227529
67	O	-9.1624294651	-4.6233163784	-0.0001151014
68	C	-6.8790172593	-6.4442934926	-0.0218773397
69	H	-7.7584928754	-6.7950776486	0.4938064632
70	H	-6.8885947702	-6.8191505620	-1.0361223869
71	H	-5.9930256101	-6.8000056479	0.4790469040

HPPI (CAM-DZ)

1	C	6.2484047125	-2.7889707593	-0.0028024299
2	C	5.5340483676	-1.5981778658	-0.0015066623
3	C	7.6397444093	-2.7873876341	0.0006567004
4	C	6.2263972121	-0.3581776200	0.0013467079
5	C	5.3551275599	0.7769022006	0.0001369131
6	H	5.7360191179	1.7929153611	0.0021900690
7	C	4.0097014913	0.5141828035	-0.0026835730
8	C	8.5259963230	0.7905816787	0.0087252796
9	H	8.1549473290	1.8095715984	0.0083195819
10	C	7.6539042566	-0.3546497593	0.0052728528
11	C	8.3511930224	-1.5921478398	0.0058228157
12	C	8.1043614448	-4.1998713169	0.0003693246
13	C	5.7811005591	-4.1987127295	-0.0052230243
14	N	6.9414430069	-4.9867361913	-0.0015311207
15	C	6.9408376386	-6.4382988732	-0.0170426094
16	H	6.0335925092	-6.7895136664	0.4762187332
17	H	6.9616293478	-6.8235622863	-1.0415150680
18	H	7.8255307520	-6.7932268854	0.5132914665
19	O	9.2455849949	-4.6214045507	0.0067395177
20	O	4.6370682910	-4.6139282939	-0.0036724786
21	Se	10.2269810581	-1.3496183938	0.0135561402
22	Se	3.6589782367	-1.3746213842	-0.0056598438
23	C	9.8527275335	0.5046579256	0.0120529308
24	H	10.6660265061	1.2182464927	0.0148305403
25	C	2.9292717372	1.4679623756	-0.0035353387

26	C	1.5829166185	1.2078147979	-0.0054773812
27	Se	3.2825731814	3.3561161190	-0.0020070511
28	H	1.2015789499	0.1920249901	-0.0067668866
29	C	0.7145810908	2.3438491104	-0.0051977426
30	C	1.4082753897	3.5841578394	-0.0030690267
31	C	-0.7145558011	2.3438308552	-0.0062939883
32	C	0.6965734727	4.7756991372	-0.0006365642
33	C	-1.4082797551	3.5841233657	-0.0055609819
34	C	-1.5828648091	1.2077745305	-0.0071139698
35	C	-0.6966035009	4.7756812319	-0.0019433046
36	C	1.1619754300	6.1857359439	0.0011518592
37	Se	-3.2825666842	3.3560434805	-0.0078644506
38	H	-1.2015034204	0.1919932592	-0.0067407699
39	C	-2.9292262796	1.4678952999	-0.0068490761
40	C	-1.1620870017	6.1856822219	-0.0011448217
41	N	-0.0000462663	6.9729949881	-0.0013785168
42	O	2.3050378041	6.6039028199	-0.0020528862
43	O	-2.3052051599	6.6036763425	-0.0068450778
44	C	0.0000029581	8.4245921646	0.0131357127
45	H	-0.8991288857	8.7768252659	-0.4940750908
46	H	0.0056097787	8.8103206232	1.0376077169
47	H	0.8931516238	8.7777748301	-0.5039620040
48	C	-4.0096515916	0.5141110942	-0.0057507592
49	C	-5.3550715801	0.7768498201	-0.0022824292
50	Se	-3.6589540320	-1.3746981309	-0.0085314983
51	H	-5.7359449588	1.7928692504	0.0000347098
52	C	-6.2263558892	-0.3582129736	-0.0001299572
53	C	-5.5340285570	-1.5982259010	-0.0028950032
54	C	-7.6538580693	-0.3546571759	0.0048611809
55	C	-6.2484000035	-2.7890061738	-0.0028812272
56	C	-8.3511679885	-1.5921392141	0.0068752345
57	C	-8.5259254807	0.7905958110	0.0079447038
58	C	-7.6397371062	-2.7873951889	0.0018798735
59	C	-5.7811648813	-4.1987800728	-0.0050097672
60	Se	-10.2269437303	-1.3495652978	0.0156938063
61	H	-8.1548538671	1.8095765103	0.0064219776
62	C	-9.8526595998	0.5047039094	0.0121834708
63	C	-8.1044209913	-4.1998468811	0.0029795479
64	N	-6.9414794760	-4.9867558783	0.0004119892
65	O	-4.6371898403	-4.6141531438	-0.0045474355
66	H	-10.6659440162	1.2183096078	0.0147671003
67	O	-9.2456976974	-4.6212105523	0.0110419459
68	C	-6.9407486701	-6.4383314355	-0.0140991334
69	H	-7.8321056759	-6.7919422958	0.5057805859
70	H	-6.9492866914	-6.8243058190	-1.0384829748
71	H	-6.0398414299	-6.7901438072	0.4902956532

HPPI (ωB97X-D3-DZ)

1	C	6.1788830649	-2.8077037009	0.0025962719
2	C	5.4658512408	-1.6304647913	0.0022595581

3	C	7.5584807620	-2.8013267070	0.0020546654
4	C	6.1536570066	-0.4095015138	0.0006021591
5	C	5.2842582828	0.7463994716	-0.0003508013
6	H	5.6805090199	1.7415187955	-0.0017443081
7	C	3.9617750468	0.5074961815	0.0006286248
8	C	8.4269763798	0.7664170059	-0.0016418061
9	H	8.0415133361	1.7652677926	-0.0028172993
10	C	7.5626423820	-0.4011544394	0.0000410695
11	C	8.2629251059	-1.6175543903	0.0011576620
12	C	8.0212595870	-4.2094506015	0.0042201270
13	C	5.7239907213	-4.2164380481	0.0052041150
14	N	6.8743423978	-4.9908595450	0.0092913461
15	C	6.8793597264	-6.4443380653	-0.0036548593
16	H	5.9872754410	-6.7976807648	0.4879704341
17	H	6.9008694362	-6.8227686597	-1.0163866719
18	H	7.7524440430	-6.7939036307	0.5236657059
19	O	9.1623197519	-4.6234719830	0.0045012540
20	O	4.5835094123	-4.6335563262	0.0065090857
21	Se	10.1578550290	-1.3570696586	0.0008447638
22	Se	3.5723247880	-1.3908392379	0.0032185369
23	C	9.7380763019	0.5099404370	-0.0020449955
24	H	10.5244463702	1.2327381676	-0.0033180432
25	C	2.8932562315	1.4847945631	0.0001318109
26	C	1.5696714643	1.2505156890	0.0000617279
27	Se	3.2892176209	3.3818750206	-0.0005209570
28	H	1.1719258373	0.2560518762	0.0003202833
29	C	0.7047484567	2.4096075348	-0.0007467567
30	C	1.3973852642	3.6293847596	-0.0011254581
31	C	-0.7047517022	2.4096073084	-0.0012887414
32	C	0.6902565061	4.8091462249	-0.0012986168
33	C	-1.3973888069	3.6293838322	-0.0021529600
34	C	-1.5696739118	1.2505136940	-0.0010564163
35	C	-0.6902573563	4.8091468612	-0.0017827161
36	C	1.1487049132	6.2171584611	-0.0026676685
37	Se	-3.2892196236	3.3818718002	-0.0032097302
38	H	-1.1719270768	0.2560505888	-0.0001658449
39	C	-2.8932579334	1.4847914676	-0.0017264198
40	C	-1.1487605257	6.2171438827	-0.0033965890
41	N	-0.0000180832	6.9949643642	-0.0071578939
42	O	2.2898449631	6.6321928195	-0.0028206053
43	O	-2.2899557215	6.6320343116	-0.0044833474
44	C	0.0001931399	8.4486625181	0.0065539529
45	H	-0.8862386026	8.7999921151	-0.4966064480
46	H	0.0074102164	8.8264658904	1.0196818679
47	H	0.8791238127	8.8005953713	-0.5092638830
48	C	-3.9617771896	0.5074932062	-0.0015939103
49	C	-5.2842560526	0.7463941880	0.0022770355
50	Se	-3.5723342241	-1.3908363499	-0.0071905630
51	H	-5.6804995416	1.7415106284	0.0059067209
52	C	-6.1536566173	-0.4095051934	0.0017167450
53	C	-5.4658575467	-1.6304644338	-0.0030731051

54	C	-7.5626367613	-0.4011616735	0.0055268414
55	C	-6.1788898799	-2.8076987947	-0.0047398543
56	C	-8.2629213341	-1.6175603052	0.0044728184
57	C	-8.4269628150	0.7664064732	0.0106797310
58	C	-7.5584825345	-2.8013265676	-0.0010304532
59	C	-5.7240566625	-4.2164468657	-0.0085041779
60	Se	-10.1578402784	-1.3570813908	0.0108632322
61	H	-8.0414959074	1.7652557713	0.0119025757
62	C	-9.7380592067	0.5099297739	0.0134476891
63	C	-8.0213178505	-4.2094355849	-0.0025489804
64	N	-6.8743787343	-4.9908688611	-0.0037180405
65	O	-4.5836350335	-4.6337040486	-0.0123620590
66	H	-10.5244238776	1.2327246141	0.0172227529
67	O	-9.1624294651	-4.6233163784	-0.0001151014
68	C	-6.8790172593	-6.4442934926	-0.0218773397
69	H	-7.7584928754	-6.7950776486	0.4938064632
70	H	-6.8885947702	-6.8191505620	-1.0361223869
71	H	-5.9930256101	-6.8000056479	0.4790469040

HPPI (B3LYP-TZ)

1	C	6.2484047125	-2.7889707593	-0.0028024299
2	C	5.5340483676	-1.5981778658	-0.0015066623
3	C	7.6397444093	-2.7873876341	0.0006567004
4	C	6.2263972121	-0.3581776200	0.0013467079
5	C	5.3551275599	0.7769022006	0.0001369131
6	H	5.7360191179	1.7929153611	0.0021900690
7	C	4.0097014913	0.5141828035	-0.0026835730
8	C	8.5259963230	0.7905816787	0.0087252796
9	H	8.1549473290	1.8095715984	0.0083195819
10	C	7.6539042566	-0.3546497593	0.0052728528
11	C	8.3511930224	-1.5921478398	0.0058228157
12	C	8.1043614448	-4.1998713169	0.0003693246
13	C	5.7811005591	-4.1987127295	-0.0052230243
14	N	6.9414430069	-4.9867361913	-0.0015311207
15	C	6.9408376386	-6.4382988732	-0.0170426094
16	H	6.0335925092	-6.7895136664	0.4762187332
17	H	6.9616293478	-6.8235622863	-1.0415150680
18	H	7.8255307520	-6.7932268854	0.5132914665
19	O	9.2455849949	-4.6214045507	0.0067395177
20	O	4.6370682910	-4.6139282939	-0.0036724786
21	Se	10.2269810581	-1.3496183938	0.0135561402
22	Se	3.6589782367	-1.3746213842	-0.0056598438
23	C	9.8527275335	0.5046579256	0.0120529308
24	H	10.6660265061	1.2182464927	0.0148305403
25	C	2.9292717372	1.4679623756	-0.0035353387
26	C	1.5829166185	1.2078147979	-0.0054773812
27	Se	3.2825731814	3.3561161190	-0.0020070511
28	H	1.2015789499	0.1920249901	-0.0067668866
29	C	0.7145810908	2.3438491104	-0.0051977426

30	C	1.4082753897	3.5841578394	-0.0030690267
31	C	-0.7145558011	2.3438308552	-0.0062939883
32	C	0.6965734727	4.7756991372	-0.0006365642
33	C	-1.4082797551	3.5841233657	-0.0055609819
34	C	-1.5828648091	1.2077745305	-0.0071139698
35	C	-0.6966035009	4.7756812319	-0.0019433046
36	C	1.1619754300	6.1857359439	0.0011518592
37	Se	-3.2825666842	3.3560434805	-0.0078644506
38	H	-1.2015034204	0.1919932592	-0.0067407699
39	C	-2.9292262796	1.4678952999	-0.0068490761
40	C	-1.1620870017	6.1856822219	-0.0011448217
41	N	-0.0000462663	6.9729949881	-0.0013785168
42	O	2.3050378041	6.6039028199	-0.0020528862
43	O	-2.3052051599	6.6036763425	-0.0068450778
44	C	0.0000029581	8.4245921646	0.0131357127
45	H	-0.8991288857	8.7768252659	-0.4940750908
46	H	0.0056097787	8.8103206232	1.0376077169
47	H	0.8931516238	8.7777748301	-0.5039620040
48	C	-4.0096515916	0.5141110942	-0.0057507592
49	C	-5.3550715801	0.7768498201	-0.0022824292
50	Se	-3.6589540320	-1.3746981309	-0.0085314983
51	H	-5.7359449588	1.7928692504	0.0000347098
52	C	-6.2263558892	-0.3582129736	-0.0001299572
53	C	-5.5340285570	-1.5982259010	-0.0028950032
54	C	-7.6538580693	-0.3546571759	0.0048611809
55	C	-6.2484000035	-2.7890061738	-0.0028812272
56	C	-8.3511679885	-1.5921392141	0.0068752345
57	C	-8.5259254807	0.7905958110	0.0079447038
58	C	-7.6397371062	-2.7873951889	0.0018798735
59	C	-5.7811648813	-4.1987800728	-0.0050097672
60	Se	-10.2269437303	-1.3495652978	0.0156938063
61	H	-8.1548538671	1.8095765103	0.0064219776
62	C	-9.8526595998	0.5047039094	0.0121834708
63	C	-8.1044209913	-4.1998468811	0.0029795479
64	N	-6.9414794760	-4.9867558783	0.0004119892
65	O	-4.6371898403	-4.6141531438	-0.0045474355
66	H	-10.6659440162	1.2183096078	0.0147671003
67	O	-9.2456976974	-4.6212105523	0.0110419459
68	C	-6.9407486701	-6.4383314355	-0.0140991334
69	H	-7.8321056759	-6.7919422958	0.5057805859
70	H	-6.9492866914	-6.8243058190	-1.0384829748
71	H	-6.0398414299	-6.7901438072	0.4902956532

HPPI (**CAM-TZ)**

1	C	6.2484047125	-2.7889707593	-0.0028024299
2	C	5.5340483676	-1.5981778658	-0.0015066623
3	C	7.6397444093	-2.7873876341	0.0006567004
4	C	6.2263972121	-0.3581776200	0.0013467079
5	C	5.3551275599	0.7769022006	0.0001369131
6	H	5.7360191179	1.7929153611	0.0021900690

7	C	4.0097014913	0.5141828035	-0.0026835730
8	C	8.5259963230	0.7905816787	0.0087252796
9	H	8.1549473290	1.8095715984	0.0083195819
10	C	7.6539042566	-0.3546497593	0.0052728528
11	C	8.3511930224	-1.5921478398	0.0058228157
12	C	8.1043614448	-4.1998713169	0.0003693246
13	C	5.7811005591	-4.1987127295	-0.0052230243
14	N	6.9414430069	-4.9867361913	-0.0015311207
15	C	6.9408376386	-6.4382988732	-0.0170426094
16	H	6.0335925092	-6.7895136664	0.4762187332
17	H	6.9616293478	-6.8235622863	-1.0415150680
18	H	7.8255307520	-6.7932268854	0.5132914665
19	O	9.2455849949	-4.6214045507	0.0067395177
20	O	4.6370682910	-4.6139282939	-0.0036724786
21	Se	10.2269810581	-1.3496183938	0.0135561402
22	Se	3.6589782367	-1.3746213842	-0.0056598438
23	C	9.8527275335	0.5046579256	0.0120529308
24	H	10.6660265061	1.2182464927	0.0148305403
25	C	2.9292717372	1.4679623756	-0.0035353387
26	C	1.5829166185	1.2078147979	-0.0054773812
27	Se	3.2825731814	3.3561161190	-0.0020070511
28	H	1.2015789499	0.1920249901	-0.0067668866
29	C	0.7145810908	2.3438491104	-0.0051977426
30	C	1.4082753897	3.5841578394	-0.0030690267
31	C	-0.7145558011	2.3438308552	-0.0062939883
32	C	0.6965734727	4.7756991372	-0.0006365642
33	C	-1.4082797551	3.5841233657	-0.0055609819
34	C	-1.5828648091	1.2077745305	-0.0071139698
35	C	-0.6966035009	4.7756812319	-0.0019433046
36	C	1.1619754300	6.1857359439	0.0011518592
37	Se	-3.2825666842	3.3560434805	-0.0078644506
38	H	-1.2015034204	0.1919932592	-0.0067407699
39	C	-2.9292262796	1.4678952999	-0.0068490761
40	C	-1.1620870017	6.1856822219	-0.0011448217
41	N	-0.0000462663	6.9729949881	-0.0013785168
42	O	2.3050378041	6.6039028199	-0.0020528862
43	O	-2.3052051599	6.6036763425	-0.0068450778
44	C	0.0000029581	8.4245921646	0.0131357127
45	H	-0.8991288857	8.7768252659	-0.4940750908
46	H	0.0056097787	8.8103206232	1.0376077169
47	H	0.8931516238	8.7777748301	-0.5039620040
48	C	-4.0096515916	0.5141110942	-0.0057507592
49	C	-5.3550715801	0.7768498201	-0.0022824292
50	Se	-3.6589540320	-1.3746981309	-0.0085314983
51	H	-5.7359449588	1.7928692504	0.0000347098
52	C	-6.2263558892	-0.3582129736	-0.0001299572
53	C	-5.5340285570	-1.5982259010	-0.0028950032
54	C	-7.6538580693	-0.3546571759	0.0048611809
55	C	-6.2484000035	-2.7890061738	-0.0028812272
56	C	-8.3511679885	-1.5921392141	0.0068752345
57	C	-8.5259254807	0.7905958110	0.0079447038

58	C	-7.6397371062	-2.7873951889	0.0018798735
59	C	-5.7811648813	-4.1987800728	-0.0050097672
60	Se	-10.2269437303	-1.3495652978	0.0156938063
61	H	-8.1548538671	1.8095765103	0.0064219776
62	C	-9.8526595998	0.5047039094	0.0121834708
63	C	-8.1044209913	-4.1998468811	0.0029795479
64	N	-6.9414794760	-4.9867558783	0.0004119892
65	O	-4.6371898403	-4.6141531438	-0.0045474355
66	H	-10.6659440162	1.2183096078	0.0147671003
67	O	-9.2456976974	-4.6212105523	0.0110419459
68	C	-6.9407486701	-6.4383314355	-0.0140991334
69	H	-7.8321056759	-6.7919422958	0.5057805859
70	H	-6.9492866914	-6.8243058190	-1.0384829748
71	H	-6.0398414299	-6.7901438072	0.4902956532

HPMI (B3LYP-DZ)

1	C	7.8061352257	-2.3380572677	0.6302777834
2	C	6.8940399408	-1.2166438517	0.5579394798
3	C	9.1384534833	-2.4089898965	0.4469426577
4	C	6.1247568188	1.0204815345	0.6061276695
5	H	6.2792307703	2.0740676482	0.7280633491
6	C	4.9249511256	0.4943428261	0.2692262728
7	C	12.2041495213	-0.3610031801	-0.2996485328
8	H	13.2172703201	-0.1647356320	-0.0121449899
9	C	10.1136498749	-1.4465368005	-0.0368973204
10	C	9.5729154472	-3.8109515561	0.7854909547
11	C	7.3232275039	-3.7101932884	1.0067999246
12	N	8.4304898502	-4.5186273721	1.1028205951
13	C	8.3949151439	-5.9296531986	1.4480889500
14	H	7.6617479576	-6.0872202768	2.2233388832
15	H	8.1361232578	-6.5318414440	0.5882283149
16	H	9.3713878277	-6.2163932216	1.8029277487
17	O	10.6991727779	-4.2589705572	0.7953612174
18	O	6.1752213366	-4.0612316524	1.1844765186
19	Se	9.8201803297	-0.3339036426	-1.5799193495
20	Se	5.0254996615	-1.4129370898	0.1638983269
21	C	11.3775360827	-1.3003686464	0.4141414921
22	H	11.7517673346	-1.8675887836	1.2409467408
23	C	7.1946003199	0.0867729079	0.7649735990
24	H	8.1808344330	0.4082485340	1.0277389165
25	C	11.6157624081	0.2348523257	-1.3475953562
26	H	12.0463026991	0.9530137034	-2.0101299675
27	C	3.6803255865	1.1965156600	0.0382452871
28	C	2.6104954331	0.7738318398	-0.6738597659
29	Se	3.3802065082	2.9546220613	0.7273709026
30	H	2.5913390900	-0.1730938830	-1.1757079800
31	C	1.5055269036	1.6773245229	-0.7411291863
32	C	1.6471650660	2.8549678051	-0.0898309282
33	H	0.6160971568	1.4316677866	-1.2843816175
34	C	0.6769415571	3.9310443156	-0.0074234484

35	C	-0.6730709852	3.9272042512	-0.0222426511
36	C	1.1262899854	5.3639274222	0.0598933999
37	C	-1.6387751723	2.8472356959	0.0533187066
38	C	-1.1279482828	5.3590313362	-0.0800506919
39	N	-0.0037929082	6.1479318059	-0.0045564911
40	O	2.2629535741	5.7733630368	0.1689969185
41	Se	-3.3933808214	2.9689362177	-0.7151541414
42	C	-1.4801676362	1.6506925846	0.6658222087
43	O	-2.2652740853	5.7670432320	-0.1878751550
44	C	-0.0143485656	7.6010712859	-0.0116858196
45	C	-3.6746937748	1.1914107547	-0.0694147305
46	C	-2.5862585797	0.7489313187	0.6015375558
47	H	-0.5772165127	1.3895135283	1.1783246184
48	H	0.9604835956	7.9437026948	0.2942606105
49	H	-0.2343247848	7.9778362790	-1.0005229947
50	H	-0.7618214800	7.9616088825	0.6779792164
51	H	-2.5532676200	-0.2122457695	1.0747522571
52	C	-4.9246545362	0.4948305433	-0.2875645556
53	C	-6.1299845284	1.0282878944	-0.5919147622
54	Se	-5.0268420837	-1.4139357029	-0.2124879337
55	H	-6.2850332455	2.0839521790	-0.6934352657
56	C	-7.2044788565	0.0991293207	-0.7453642507
57	C	-6.9023205169	-1.2080504303	-0.5662496243
58	H	-8.1950570986	0.4265258556	-0.9832880057
59	C	-7.8177509822	-2.3266704091	-0.6395319950
60	C	-9.1463273454	-2.3985753392	-0.4310967716
61	C	-7.3449915916	-3.6929812137	-1.0486764910
62	C	-10.1098776379	-1.4428742422	0.0881570562
63	C	-9.5900752669	-3.7939117275	-0.7847015122
64	N	-8.4554580175	-4.4980144712	-1.1361003161
65	O	-6.2013665207	-4.0428819811	-1.2545669938
66	Se	-9.7834795878	-0.3564334946	1.6431856249
67	C	-11.3821307817	-1.2869475338	-0.3352679954
68	O	-10.7170939579	-4.2400940515	-0.7793440772
69	C	-8.4293278399	-5.9031952900	-1.5052318064
70	C	-11.5826753634	0.2178780559	1.4571219449
71	C	-12.1927085049	-0.3587646913	0.4108003275
72	H	-11.7737620918	-1.8394363607	-1.1639821211
73	H	-7.7119993843	-6.0490277311	-2.2974100458
74	H	-8.1546076543	-6.5199105572	-0.6607460113
75	H	-9.4131826538	-6.1825510859	-1.8452599451
76	H	-11.9987591578	0.9250211483	2.1404195620
77	H	-13.2110105297	-0.1561851433	0.1469557242

HPMI (CAM-DZ)

1	C	8.3805685642	-2.1191037517	0.5482377954
2	C	7.3898234422	-1.0838044941	0.4659905537
3	C	9.7512892813	-2.1198999853	0.4108847973
4	C	6.3976594723	1.0725365758	0.4708750121
5	H	6.4306879999	2.1562015166	0.5262110631

6	C	5.2140487975	0.3799386878	0.2700899193
7	C	12.8234412326	-0.1786282262	-0.5499794871
8	H	13.8926132170	-0.0640953115	-0.4053602435
9	C	10.7005063311	-1.1523523118	-0.0881602616
10	C	10.2388769823	-3.5030280785	0.7858817223
11	C	7.9579021311	-3.5214917372	0.8981168746
12	N	9.1059631049	-4.2735764597	1.0616060205
13	C	9.1283282450	-5.6816408116	1.4126234020
14	H	8.6321125665	-5.8427584786	2.3730137695
15	H	8.6191488295	-6.2761314366	0.6493180505
16	H	10.1737385217	-5.9833188898	1.4792422613
17	O	11.3791611198	-3.9193855804	0.8435085038
18	O	6.8206988903	-3.9461617329	1.0144785019
19	Se	10.2934881950	0.1396767881	-1.4390857971
20	Se	5.5371623559	-1.4937037850	0.2679522225
21	C	12.0538436646	-1.1258655532	0.1826981852
22	H	12.4922235247	-1.8085965797	0.8990760259
23	C	7.5606934379	0.2877887898	0.5729065564
24	H	8.5354153778	0.7282640192	0.7412358938
25	C	12.1184064690	0.5678248710	-1.4483272542
26	H	12.4937993147	1.3378763743	-2.1085828691
27	C	3.8943732410	0.9225105102	0.1183828847
28	C	2.7640227976	0.2814213155	-0.3631875894
29	Se	3.4928882299	2.7283741574	0.5575875324
30	H	2.7894273801	-0.7453189902	-0.7151398771
31	C	1.5882361351	1.0507815612	-0.4032551811
32	C	1.6891649657	2.3643491303	0.0381097481
33	H	0.6639043644	0.6540230745	-0.8029693434
34	C	0.6909191036	3.3905977924	0.0658700338
35	C	-0.6923028752	3.3905119128	-0.0524909991
36	C	1.1235792832	4.8267937337	0.1789174398
37	C	-1.6909589878	2.3652192795	-0.0233060727
38	C	-1.1231818453	4.8266866132	-0.1733097614
39	N	-0.0004477782	5.6221343265	-0.0004311428
40	O	2.2481015278	5.2488737194	0.3888726712
41	Se	-3.4922709651	2.7286767841	-0.5516138298
42	C	-1.5928546717	1.0537581393	0.4249552958
43	O	-2.2447717959	5.2532668620	-0.3898142369
44	C	-0.0049800394	7.0738369884	-0.0025724800
45	C	-3.8966011772	0.9245291577	-0.1071385902
46	C	-2.7689304437	0.2851093572	0.3830841021
47	H	-0.6704596666	0.6584063924	0.8305562714
48	H	1.0323899072	7.4042953714	0.0509730693
49	H	-0.4705122780	7.4454459468	-0.9182417180
50	H	-0.5591878715	7.4610165295	0.8569425793
51	H	-2.7965469002	-0.7401211208	0.7392634905
52	C	-5.2151593361	0.3807629844	-0.2641294116
53	C	-6.3976109671	1.0712217991	-0.4785180061
54	Se	-5.5374150361	-1.4930453405	-0.2513084570
55	H	-6.4307794740	2.1544300324	-0.5420873439
56	C	-7.5594298881	0.2850679761	-0.5842556491

57	C	-7.3885459638	-1.0856611673	-0.4672737346
58	H	-8.5330418144	0.7238230193	-0.7632993273
59	C	-8.3779361684	-2.1223489846	-0.5497660720
60	C	-9.7491810146	-2.1238475637	-0.4181957677
61	C	-7.9523511568	-3.5258315526	-0.8915121449
62	C	-10.7010698183	-1.1550529682	0.0731183983
63	C	-10.2338872160	-3.5091132129	-0.7891560969
64	N	-9.0990854873	-4.2798043886	-1.0563059194
65	O	-6.8143001881	-3.9499988147	-1.0009309553
66	Se	-10.2987330861	0.1454917347	1.4170104741
67	C	-12.0539442781	-1.1326301485	-0.2004371857
68	O	-11.3735530978	-3.9267708223	-0.8497707331
69	C	-9.1185541431	-5.6892585285	-1.4017955851
70	C	-12.1243097813	0.5710833912	1.4198220137
71	C	-12.8264982075	-0.1822298832	0.5249702518
72	H	-12.4897884306	-1.8205523929	-0.9133888638
73	H	-8.6201520736	-5.8535373418	-2.3605380768
74	H	-8.6099975512	-6.2797979199	-0.6350346470
75	H	-10.1633538213	-5.9928322552	-1.4693852901
76	H	-12.5021828288	1.3445964106	2.0745925964
77	H	-13.8956019826	-0.0704225328	0.3777309396

HPMI (ωB97X-D3-DZ)

1	C	7.8061352257	-2.3380572677	0.6302777834
2	C	6.8940399408	-1.2166438517	0.5579394798
3	C	9.1384534833	-2.4089898965	0.4469426577
4	C	6.1247568188	1.0204815345	0.6061276695
5	H	6.2792307703	2.0740676482	0.7280633491
6	C	4.9249511256	0.4943428261	0.2692262728
7	C	12.2041495213	-0.3610031801	-0.2996485328
8	H	13.2172703201	-0.1647356320	-0.0121449899
9	C	10.1136498749	-1.4465368005	-0.0368973204
10	C	9.5729154472	-3.8109515561	0.7854909547
11	C	7.3232275039	-3.7101932884	1.0067999246
12	N	8.4304898502	-4.5186273721	1.1028205951
13	C	8.3949151439	-5.9296531986	1.4480889500
14	H	7.6617479576	-6.0872202768	2.2233388832
15	H	8.1361232578	-6.5318414440	0.5882283149
16	H	9.3713878277	-6.2163932216	1.8029277487
17	O	10.6991727779	-4.2589705572	0.7953612174
18	O	6.1752213366	-4.0612316524	1.1844765186
19	Se	9.8201803297	-0.3339036426	-1.5799193495
20	Se	5.0254996615	-1.4129370898	0.1638983269
21	C	11.3775360827	-1.3003686464	0.4141414921
22	H	11.7517673346	-1.8675887836	1.2409467408
23	C	7.1946003199	0.0867729079	0.7649735990
24	H	8.1808344330	0.4082485340	1.0277389165
25	C	11.6157624081	0.2348523257	-1.3475953562
26	H	12.0463026991	0.9530137034	-2.0101299675
27	C	3.6803255865	1.1965156600	0.0382452871
28	C	2.6104954331	0.7738318398	-0.6738597659

29	Se	3.3802065082	2.9546220613	0.7273709026
30	H	2.5913390900	-0.1730938830	-1.1757079800
31	C	1.5055269036	1.6773245229	-0.7411291863
32	C	1.6471650660	2.8549678051	-0.0898309282
33	H	0.6160971568	1.4316677866	-1.2843816175
34	C	0.6769415571	3.9310443156	-0.0074234484
35	C	-0.6730709852	3.9272042512	-0.0222426511
36	C	1.1262899854	5.3639274222	0.0598933999
37	C	-1.6387751723	2.8472356959	0.0533187066
38	C	-1.1279482828	5.3590313362	-0.0800506919
39	N	-0.0037929082	6.1479318059	-0.0045564911
40	O	2.2629535741	5.7733630368	0.1689969185
41	Se	-3.3933808214	2.9689362177	-0.7151541414
42	C	-1.4801676362	1.6506925846	0.6658222087
43	O	-2.2652740853	5.7670432320	-0.1878751550
44	C	-0.0143485656	7.6010712859	-0.0116858196
45	C	-3.6746937748	1.1914107547	-0.0694147305
46	C	-2.5862585797	0.7489313187	0.6015375558
47	H	-0.5772165127	1.3895135283	1.1783246184
48	H	0.9604835956	7.9437026948	0.2942606105
49	H	-0.2343247848	7.9778362790	-1.0005229947
50	H	-0.7618214800	7.9616088825	0.6779792164
51	H	-2.5532676200	-0.2122457695	1.0747522571
52	C	-4.9246545362	0.4948305433	-0.2875645556
53	C	-6.1299845284	1.0282878944	-0.5919147622
54	Se	-5.0268420837	-1.4139357029	-0.2124879337
55	H	-6.2850332455	2.0839521790	-0.6934352657
56	C	-7.2044788565	0.0991293207	-0.7453642507
57	C	-6.9023205169	-1.2080504303	-0.5662496243
58	H	-8.1950570986	0.4265258556	-0.9832880057
59	C	-7.8177509822	-2.3266704091	-0.6395319950
60	C	-9.1463273454	-2.3985753392	-0.4310967716
61	C	-7.3449915916	-3.6929812137	-1.0486764910
62	C	-10.1098776379	-1.4428742422	0.0881570562
63	C	-9.5900752669	-3.7939117275	-0.7847015122
64	N	-8.4554580175	-4.4980144712	-1.1361003161
65	O	-6.2013665207	-4.0428819811	-1.2545669938
66	Se	-9.7834795878	-0.3564334946	1.6431856249
67	C	-11.3821307817	-1.2869475338	-0.3352679954
68	O	-10.7170939579	-4.2400940515	-0.7793440772
69	C	-8.4293278399	-5.9031952900	-1.5052318064
70	C	-11.5826753634	0.2178780559	1.4571219449
71	C	-12.1927085049	-0.3587646913	0.4108003275
72	H	-11.7737620918	-1.8394363607	-1.1639821211
73	H	-7.7119993843	-6.0490277311	-2.2974100458
74	H	-8.1546076543	-6.5199105572	-0.6607460113
75	H	-9.4131826538	-6.1825510859	-1.8452599451
76	H	-11.9987591578	0.9250211483	2.1404195620
77	H	-13.2110105297	-0.1561851433	0.1469557242

HPMI (B3LYP-TZ)

1	C	8.3805685642	-2.1191037517	0.5482377954
2	C	7.3898234422	-1.0838044941	0.4659905537
3	C	9.7512892813	-2.1198999853	0.4108847973
4	C	6.3976594723	1.0725365758	0.4708750121
5	H	6.4306879999	2.1562015166	0.5262110631
6	C	5.2140487975	0.3799386878	0.2700899193
7	C	12.8234412326	-0.1786282262	-0.5499794871
8	H	13.8926132170	-0.0640953115	-0.4053602435
9	C	10.7005063311	-1.1523523118	-0.0881602616
10	C	10.2388769823	-3.5030280785	0.7858817223
11	C	7.9579021311	-3.5214917372	0.8981168746
12	N	9.1059631049	-4.2735764597	1.0616060205
13	C	9.1283282450	-5.6816408116	1.4126234020
14	H	8.6321125665	-5.8427584786	2.3730137695
15	H	8.6191488295	-6.2761314366	0.6493180505
16	H	10.1737385217	-5.9833188898	1.4792422613
17	O	11.3791611198	-3.9193855804	0.8435085038
18	O	6.8206988903	-3.9461617329	1.0144785019
19	Se	10.2934881950	0.1396767881	-1.4390857971
20	Se	5.5371623559	-1.4937037850	0.2679522225
21	C	12.0538436646	-1.1258655532	0.1826981852
22	H	12.4922235247	-1.8085965797	0.8990760259
23	C	7.5606934379	0.2877887898	0.5729065564
24	H	8.5354153778	0.7282640192	0.7412358938
25	C	12.1184064690	0.5678248710	-1.4483272542
26	H	12.4937993147	1.3378763743	-2.1085828691
27	C	3.8943732410	0.9225105102	0.1183828847
28	C	2.7640227976	0.2814213155	-0.3631875894
29	Se	3.4928882299	2.7283741574	0.5575875324
30	H	2.7894273801	-0.7453189902	-0.7151398771
31	C	1.5882361351	1.0507815612	-0.4032551811
32	C	1.6891649657	2.3643491303	0.0381097481
33	H	0.6639043644	0.6540230745	-0.8029693434
34	C	0.6909191036	3.3905977924	0.0658700338
35	C	-0.6923028752	3.3905119128	-0.0524909991
36	C	1.1235792832	4.8267937337	0.1789174398
37	C	-1.6909589878	2.3652192795	-0.0233060727
38	C	-1.1231818453	4.8266866132	-0.1733097614
39	N	-0.0004477782	5.6221343265	-0.0004311428
40	O	2.2481015278	5.2488737194	0.3888726712
41	Se	-3.4922709651	2.7286767841	-0.5516138298
42	C	-1.5928546717	1.0537581393	0.4249552958
43	O	-2.2447717959	5.2532668620	-0.3898142369
44	C	-0.0049800394	7.0738369884	-0.0025724800
45	C	-3.8966011772	0.9245291577	-0.1071385902
46	C	-2.7689304437	0.2851093572	0.3830841021
47	H	-0.6704596666	0.6584063924	0.8305562714
48	H	1.0323899072	7.4042953714	0.0509730693
49	H	-0.4705122780	7.4454459468	-0.9182417180
50	H	-0.5591878715	7.4610165295	0.8569425793

51	H	-2.7965469002	-0.7401211208	0.7392634905
52	C	-5.2151593361	0.3807629844	-0.2641294116
53	C	-6.3976109671	1.0712217991	-0.4785180061
54	Se	-5.5374150361	-1.4930453405	-0.2513084570
55	H	-6.4307794740	2.1544300324	-0.5420873439
56	C	-7.5594298881	0.2850679761	-0.5842556491
57	C	-7.3885459638	-1.0856611673	-0.4672737346
58	H	-8.5330418144	0.7238230193	-0.7632993273
59	C	-8.3779361684	-2.1223489846	-0.5497660720
60	C	-9.7491810146	-2.1238475637	-0.4181957677
61	C	-7.9523511568	-3.5258315526	-0.8915121449
62	C	-10.7010698183	-1.1550529682	0.0731183983
63	C	-10.2338872160	-3.5091132129	-0.7891560969
64	N	-9.0990854873	-4.2798043886	-1.0563059194
65	O	-6.8143001881	-3.9499988147	-1.0009309553
66	Se	-10.2987330861	0.1454917347	1.4170104741
67	C	-12.0539442781	-1.1326301485	-0.2004371857
68	O	-11.3735530978	-3.9267708223	-0.8497707331
69	C	-9.1185541431	-5.6892585285	-1.4017955851
70	C	-12.1243097813	0.5710833912	1.4198220137
71	C	-12.8264982075	-0.1822298832	0.5249702518
72	H	-12.4897884306	-1.8205523929	-0.9133888638
73	H	-8.6201520736	-5.8535373418	-2.3605380768
74	H	-8.6099975512	-6.2797979199	-0.6350346470
75	H	-10.1633538213	-5.9928322552	-1.4693852901
76	H	-12.5021828288	1.3445964106	2.0745925964
77	H	-13.8956019826	-0.0704225328	0.3777309396

HPMI (CAM-TZ)

1	C	8.3805685642	-2.1191037517	0.5482377954
2	C	7.3898234422	-1.0838044941	0.4659905537
3	C	9.7512892813	-2.1198999853	0.4108847973
4	C	6.3976594723	1.0725365758	0.4708750121
5	H	6.4306879999	2.1562015166	0.5262110631
6	C	5.2140487975	0.3799386878	0.2700899193
7	C	12.8234412326	-0.1786282262	-0.5499794871
8	H	13.8926132170	-0.0640953115	-0.4053602435
9	C	10.7005063311	-1.1523523118	-0.0881602616
10	C	10.2388769823	-3.5030280785	0.7858817223
11	C	7.9579021311	-3.5214917372	0.8981168746
12	N	9.1059631049	-4.2735764597	1.0616060205
13	C	9.1283282450	-5.6816408116	1.4126234020
14	H	8.6321125665	-5.8427584786	2.3730137695
15	H	8.6191488295	-6.2761314366	0.6493180505
16	H	10.1737385217	-5.9833188898	1.4792422613
17	O	11.3791611198	-3.9193855804	0.8435085038
18	O	6.8206988903	-3.9461617329	1.0144785019
19	Se	10.2934881950	0.1396767881	-1.4390857971
20	Se	5.5371623559	-1.4937037850	0.2679522225
21	C	12.0538436646	-1.1258655532	0.1826981852
22	H	12.4922235247	-1.8085965797	0.8990760259

23	C	7.5606934379	0.2877887898	0.5729065564
24	H	8.5354153778	0.7282640192	0.7412358938
25	C	12.1184064690	0.5678248710	-1.4483272542
26	H	12.4937993147	1.3378763743	-2.1085828691
27	C	3.8943732410	0.9225105102	0.1183828847
28	C	2.7640227976	0.2814213155	-0.3631875894
29	Se	3.4928882299	2.7283741574	0.5575875324
30	H	2.7894273801	-0.7453189902	-0.7151398771
31	C	1.5882361351	1.0507815612	-0.4032551811
32	C	1.6891649657	2.3643491303	0.0381097481
33	H	0.6639043644	0.6540230745	-0.8029693434
34	C	0.6909191036	3.3905977924	0.0658700338
35	C	-0.6923028752	3.3905119128	-0.0524909991
36	C	1.1235792832	4.8267937337	0.1789174398
37	C	-1.6909589878	2.3652192795	-0.0233060727
38	C	-1.1231818453	4.8266866132	-0.1733097614
39	N	-0.0004477782	5.6221343265	-0.0004311428
40	O	2.2481015278	5.2488737194	0.3888726712
41	Se	-3.4922709651	2.7286767841	-0.5516138298
42	C	-1.5928546717	1.0537581393	0.4249552958
43	O	-2.2447717959	5.2532668620	-0.3898142369
44	C	-0.0049800394	7.0738369884	-0.0025724800
45	C	-3.8966011772	0.9245291577	-0.1071385902
46	C	-2.7689304437	0.2851093572	0.3830841021
47	H	-0.6704596666	0.6584063924	0.8305562714
48	H	1.0323899072	7.4042953714	0.0509730693
49	H	-0.4705122780	7.4454459468	-0.9182417180
50	H	-0.5591878715	7.4610165295	0.8569425793
51	H	-2.7965469002	-0.7401211208	0.7392634905
52	C	-5.2151593361	0.3807629844	-0.2641294116
53	C	-6.3976109671	1.0712217991	-0.4785180061
54	Se	-5.5374150361	-1.4930453405	-0.2513084570
55	H	-6.4307794740	2.1544300324	-0.5420873439
56	C	-7.5594298881	0.2850679761	-0.5842556491
57	C	-7.3885459638	-1.0856611673	-0.4672737346
58	H	-8.5330418144	0.7238230193	-0.7632993273
59	C	-8.3779361684	-2.1223489846	-0.5497660720
60	C	-9.7491810146	-2.1238475637	-0.4181957677
61	C	-7.9523511568	-3.5258315526	-0.8915121449
62	C	-10.7010698183	-1.1550529682	0.0731183983
63	C	-10.2338872160	-3.5091132129	-0.7891560969
64	N	-9.0990854873	-4.2798043886	-1.0563059194
65	O	-6.8143001881	-3.9499988147	-1.0009309553
66	Se	-10.2987330861	0.1454917347	1.4170104741
67	C	-12.0539442781	-1.1326301485	-0.2004371857
68	O	-11.3735530978	-3.9267708223	-0.8497707331
69	C	-9.1185541431	-5.6892585285	-1.4017955851
70	C	-12.1243097813	0.5710833912	1.4198220137
71	C	-12.8264982075	-0.1822298832	0.5249702518
72	H	-12.4897884306	-1.8205523929	-0.9133888638
73	H	-8.6201520736	-5.8535373418	-2.3605380768

74	H	-8.6099975512	-6.2797979199	-0.6350346470
75	H	-10.1633538213	-5.9928322552	-1.4693852901
76	H	-12.5021828288	1.3445964106	2.0745925964
77	H	-13.8956019826	-0.0704225328	0.3777309396

S-HPPI (B3LYP-DZ)

1	C	6.1788839328	-2.7528403508	0.0025551316
2	C	5.4658518510	-1.5756016084	0.0021817091
3	C	7.5584815895	-2.7464631246	0.0019212441
4	C	6.1536572593	-0.3546383174	0.0003912364
5	C	5.2842582413	0.8012624261	-0.0005864996
6	H	5.6805086845	1.7963817257	-0.0020775275
7	C	3.9617751215	0.5623589468	0.0004980308
8	C	8.4269762414	0.8212804855	-0.0020881128
9	H	8.0415129194	1.8201311099	-0.0033093635
10	C	7.5626425925	-0.3462910063	-0.0002642312
11	C	8.2629256340	-1.5626907367	0.0008927185
12	C	8.0212608410	-4.1545867698	0.0041565785
13	C	5.7239920473	-4.1615745973	0.0052939708
14	N	6.8743441492	-4.9359955741	0.0093600031
15	C	6.8793609085	-6.3894750152	-0.0034826185
16	H	5.9873094184	-6.7427827399	0.4882273117
17	H	6.9008032876	-6.7679780106	-1.0161888017
18	H	7.7524803917	-6.7390027070	0.5238048248
19	O	9.1623211054	-4.5686079059	0.0043913581
20	O	4.5835109116	-4.5786930052	0.0067046734
21	S	10.1578554796	-1.3022056557	0.0004350714
22	S	3.5723254180	-1.3359763591	0.0032495875
23	C	9.7380761854	0.5648041460	-0.0025602312
24	H	10.5244460218	1.2876019383	-0.0039372962
25	C	2.8932560789	1.5396570805	0.0000024640
26	C	1.5696713571	1.3053779419	0.0000372279
27	S	3.2892170422	3.4367375643	-0.0008122922
28	H	1.1719259483	0.3109140720	0.0003933571
29	C	0.7047480643	2.4644695570	-0.0007965582
30	C	1.3973845996	3.6842468877	-0.0013085701
31	C	-0.7047521275	2.4644690149	-0.0012447275
32	C	0.6902555941	4.8640081987	-0.0015190108
33	C	-1.3973895337	3.6842453377	-0.0021500536
34	C	-1.5696740865	1.3053752501	-0.0008719633
35	C	-0.6902582974	4.8640085291	-0.0019112240
36	C	1.1487036257	6.2720204234	-0.0030192436
37	S	-3.2892203667	3.4367328591	-0.0030632086
38	H	-1.1719269930	0.3109122893	0.0000632343
39	C	-2.8932581969	1.5396527152	-0.0014706198
40	C	-1.1487618565	6.2720053416	-0.0035952452
41	N	-0.0000198235	7.0498257779	-0.0074886206
42	O	2.2898435795	6.6870549941	-0.0032778072
43	O	-2.2899572056	6.6868954675	-0.0046357093
44	C	0.0001920198	8.5035249084	0.0061192787
45	H	-0.8862732815	8.8548183565	-0.4970072381

46	H	0.0074764533	8.8814007156	1.0192196971
47	H	0.8790882874	8.8554210546	-0.5097822176
48	C	-3.9617772452	0.5623542559	-0.0011971175
49	C	-5.2842558957	0.8012552539	0.0027447711
50	S	-3.5723342711	-1.3359756191	-0.0066839686
51	H	-5.6804993426	1.7963718735	0.0063296836
52	C	-6.1536562632	-0.3546443354	0.0023249895
53	C	-5.4658572672	-1.5756037800	-0.0024233465
54	C	-7.5626361521	-0.3463008199	0.0062282701
55	C	-6.1788894729	-2.7528383971	-0.0039584694
56	C	-8.2629205488	-1.5626996614	0.0053078251
57	C	-8.4269620960	0.8212675224	0.0113552124
58	C	-7.5584818788	-2.7464661759	-0.0001576990
59	C	-5.7240562236	-4.1615866443	-0.0076523467
60	S	-10.1578391159	-1.3022206631	0.0118057403
61	H	-8.0414953088	1.8201169811	0.0124809870
62	C	-9.7380582489	0.5647907640	0.0142287732
63	C	-8.0213170116	-4.1545753891	-0.0015447463
64	N	-6.8743778184	-4.9360085215	-0.0027342767
65	O	-4.5836347699	-4.5788438779	-0.0115563013
66	H	-10.5244228123	1.2875857178	0.0180045002
67	O	-9.1624283783	-4.5684562317	0.0009946752
68	C	-6.8790172596	-6.3894344485	-0.0207893532
69	H	-7.7584584796	-6.7401819072	0.4949780642
70	H	-6.8886622025	-6.7643640332	-1.0350069573
71	H	-5.9929921996	-6.7451106148	0.4801013490

S-HPMI (B3LYP-DZ)

1	C	7.8186619926	-2.2344531488	0.4712223308
2	C	6.9051287285	-1.1131525601	0.4177372519
3	C	9.1469602421	-2.3051757108	0.2606582663
4	C	6.1366951766	1.1238605236	0.4820097708
5	H	6.2934913200	2.1774485298	0.6009277811
6	C	4.9303183033	0.5976113261	0.1696400947
7	C	12.1964690946	-0.2566547826	-0.5481667820
8	H	13.2152334129	-0.0602944054	-0.2814181052
9	C	10.1119264551	-1.3425140821	-0.2428771197
10	C	9.5884434401	-3.7071312642	0.5900241034
11	C	7.3437403893	-3.6067138727	0.8573259781
12	N	8.4528427676	-4.4150122842	0.9305457332
13	C	8.4245263486	-5.8260970313	1.2762445858
14	H	7.7073932892	-5.9838853871	2.0663059606
15	H	8.1482779569	-6.4281857323	0.4217621359
16	H	9.4080919162	-6.1127597553	1.6109871332
17	O	10.7147265092	-4.1549985145	0.5767803645
18	O	6.1996559629	-3.9579363220	1.0583948265
19	S	9.7868039761	-0.2296790741	-1.7793951050
20	S	5.0289444584	-1.3096383570	0.0619730645
21	C	11.3847558952	-1.1962445963	0.1822345595
22	H	11.7758986376	-1.7635433606	1.0011204491
23	C	7.2096884399	0.1902726332	0.6187871577

24	H	8.2010489786	0.5118413032	0.8613727896
25	C	11.5866874817	0.3392848587	-1.5837622879
26	H	12.0034883802	1.0576086490	-2.2548515227
27	C	3.6811346360	1.2996509449	-0.0357185574
28	C	2.5970165900	0.8769331241	-0.7258558495
29	S	3.3949426224	3.0576084639	0.6596828994
30	H	2.5677234665	-0.0699165379	-1.2273581431
31	C	1.4907830994	1.7802859394	-0.7703621390
32	C	1.6455591414	2.9578464048	-0.1219099686
33	H	0.5904585015	1.5345932899	-1.2953444729
34	C	0.6770815692	4.0337779264	-0.0194997185
35	C	-0.6729510877	4.0297564425	-0.0066984521
36	C	1.1275227090	5.4667116105	0.0388389612
37	C	-1.6367638668	2.9496446327	0.0884308349
38	C	-1.1291060446	5.4615306456	-0.0549603788
39	N	-0.0037463325	6.2505722691	-0.0023536645
40	O	2.2661260025	5.8762848224	0.1247315591
41	S	-3.4067395751	3.0712267752	-0.6439667986
42	C	-1.4655001933	1.7530271451	0.6973706106
43	O	-2.2684538607	5.8694046359	-0.1394302851
44	C	-0.0146387074	7.7037113984	-0.0090338292
45	C	-3.6745471998	1.2935618650	0.0071089875
46	C	-2.5725549388	0.8511254298	0.6555835463
47	H	-0.5522187770	1.4918906723	1.1912519203
48	H	0.9662028364	8.0464275316	0.2769605078
49	H	-0.2548481785	8.0806014104	-0.9931037884
50	H	-0.7478942023	8.0640391716	0.6958358420
51	H	-2.5297623385	-0.1101213078	1.1278710165
52	C	-4.9286166267	0.5968457316	-0.1855350995
53	C	-6.1399915118	1.1301867177	-0.4650784132
54	S	-5.0289932840	-1.3119461472	-0.1086880677
55	H	-6.2972249098	2.1858457867	-0.5632374047
56	C	-7.2172767125	0.2009059692	-0.5966624109
57	C	-6.9113436723	-1.1062606989	-0.4239751506
58	H	-8.2125584877	0.5282049619	-0.8142193349
59	C	-7.8279331002	-2.2249937627	-0.4786930822
60	C	-9.1519577722	-2.2971121809	-0.2431335011
61	C	-7.3634612234	-3.5911760739	-0.8976413400
62	C	-10.1048107052	-1.3416236228	0.2958759553
63	C	-9.6026613276	-3.6924535193	-0.5878087410
64	N	-8.4753767596	-4.3963467523	-0.9624577971
65	O	-6.2242410319	-3.9408889157	-1.1269403429
66	S	-9.7468131774	-0.2553821725	1.8440750112
67	C	-11.3854805583	-1.1858037189	-0.1014084007
68	O	-10.7292752637	-4.1387900649	-0.5594675120
69	C	-8.4566168874	-5.8014661355	-1.3322706725
70	C	-11.5495151279	0.3187136472	1.6949490353
71	C	-12.1807492020	-0.2578481400	0.6612342449
72	H	-11.7939099932	-1.7382159636	-0.9220253623
73	H	-7.7556252437	-5.9470767952	-2.1389811709
74	H	-8.1645961070	-6.4182763434	-0.4936800747

75	H	-9.4471848328	-6.0809025440	-1.6521449131
76	H	-11.9516271736	1.0256930100	2.3867284754
77	H	-13.2042626515	-0.0553658425	0.4183091545

O-HPPI (B3LYP-DZ)

1	C	6.1788845488	-2.7181199319	0.0024843054
2	C	5.4658522723	-1.5408813107	0.0021005694
3	C	7.5584821749	-2.7117425212	0.0017887572
4	C	6.1536574149	-0.3199179811	0.0002358422
5	C	5.2842581788	0.8359825961	-0.0007444819
6	H	5.6805084043	1.8311019013	-0.0022887118
7	C	3.9617751449	0.5970789571	0.0004074744
8	C	8.4269761059	0.8560010738	-0.0023867542
9	H	8.0415125783	1.8548515960	-0.0036265308
10	C	7.5626427163	-0.3115704819	-0.0004826651
11	C	8.2629259932	-1.5279700649	0.0006865651
12	C	8.0212617392	-4.1198660161	0.0040537986
13	C	5.7239929995	-4.1268541480	0.0052937369
14	N	6.8743453988	-4.9012748062	0.0093362158
15	C	6.8793618069	-6.3547547045	-0.0034546942
16	H	5.9873322669	-6.7080449935	0.4883075820
17	H	6.9007591481	-6.7332938820	-1.0161483061
18	H	7.7525048221	-6.7042634242	0.5238063578
19	O	9.1623220758	-4.5338869722	0.0042525609
20	O	4.5835119910	-4.5439726765	0.0067701289
21	O	10.1578557770	-1.2674847159	0.0001352301
22	O	3.5723258524	-1.3012563078	0.0032442035
23	C	9.7380760665	0.5995249145	-0.0029080909
24	H	10.5244457311	1.3223227752	-0.0043459993
25	C	2.8932559330	1.5743769120	-0.0000754321
26	C	1.5696712496	1.3400975760	0.0000266416
27	O	3.2892165716	3.4714574249	-0.0009756058
28	H	1.1719260080	0.3456336597	0.0004360159
29	C	0.7047477446	2.4991890307	-0.0008100459
30	C	1.3973840713	3.7189664463	-0.0013964851
31	C	-0.7047524657	2.4991882609	-0.0011954508
32	C	0.6902548781	4.8987276428	-0.0016175923
33	C	-1.3973900967	3.7189644466	-0.0021135185
34	C	-1.5696742314	1.3400943803	-0.0007427559
35	C	-0.6902590296	4.8987277519	-0.0019483319
36	C	1.1487026287	6.3067398819	-0.0031885500
37	O	-3.2892209310	3.4714516513	-0.0029335872
38	H	-1.1719269457	0.3456315132	0.0002102639
39	C	-2.8932584027	1.5743716250	-0.0012908449
40	C	-1.1487628769	6.3067244344	-0.0036622460
41	N	-0.0000211364	7.0845449036	-0.0076345670
42	O	2.2898425069	6.7217746144	-0.0035127578
43	O	-2.2899583341	6.7216143515	-0.0046667178
44	C	0.0001910922	8.5382445195	0.0059213800
45	H	-0.8862966656	8.8895198568	-0.4971782143

46	H	0.0075205812	8.9161565272	1.0190079703
47	H	0.8790643326	8.8901223635	-0.5100318260
48	C	-3.9617772894	0.5970730156	-0.0009348416
49	C	-5.2842557994	0.8359739557	0.0030574001
50	O	-3.5723342719	-1.3012569957	-0.0063712042
51	H	-5.6804992372	1.8310906433	0.0066244001
52	C	-6.1536560093	-0.3199257784	0.0027176345
53	C	-5.4658570401	-1.5408852886	-0.0020177022
54	C	-7.5626357242	-0.3115823351	0.0066833582
55	C	-6.1788891347	-2.7181200669	-0.0034790097
56	C	-8.2629199766	-1.5279813139	0.0058375603
57	C	-8.4269616161	0.8559860599	0.0118070695
58	C	-7.5584813710	-2.7117479171	0.0003829656
59	C	-5.7240558366	-4.1268683769	-0.0071428038
60	O	-10.1578382920	-1.2675023681	0.0124105480
61	H	-8.0414949308	1.8548356161	0.0128799891
62	C	-9.7380576008	0.5995092075	0.0147481772
63	C	-8.0213163513	-4.1198572485	-0.0009331580
64	N	-6.8743770937	-4.9012902506	-0.0021458393
65	O	-4.5836344945	-4.5441255784	-0.0110826317
66	H	-10.5244221050	1.3223041776	0.0185330940
67	O	-9.1624275411	-4.5337381714	0.0016718652
68	C	-6.8790171182	-6.3547168226	-0.0201487761
69	H	-7.7584353173	-6.7054459839	0.4956703344
70	H	-6.8887071669	-6.7296826480	-1.0343525523
71	H	-5.9929697007	-6.7103749580	0.4807151799

O-HPMI (B3LYP-DZ)

1	C	7.8246185044	-2.1691499646	0.3765868531
2	C	6.9104036653	-1.0479263138	0.3343087116
3	C	9.1502621836	-2.2397388750	0.1498685539
4	C	6.1426129573	1.1890137852	0.4080992170
5	H	6.3007522237	2.2426066941	0.5251810914
6	C	4.9325702666	0.6626812714	0.1103930455
7	C	12.1895227294	-0.1908865289	-0.6958498344
8	H	13.2114405188	0.0055439856	-0.4415034657
9	C	10.1089448471	-1.2769536629	-0.3652986758
10	C	9.5958437957	-3.6416801814	0.4737300104
11	C	7.3545510299	-3.5414819574	0.7683361622
12	N	8.4645332039	-4.3496875901	0.8279915805
13	C	8.4405495501	-5.7608009662	1.1739012566
14	H	7.7330979027	-5.9187125999	1.9726190094
15	H	8.1539760004	-6.3628495653	0.3227976672
16	H	9.4281409971	-6.0474018086	1.4966277772
17	O	10.7219215773	-4.0894465974	0.4467468900
18	O	6.2130289916	-3.8928210096	0.9832858584
19	O	9.7650505405	-0.1640313470	-1.8976610758
20	O	5.0300461755	-1.2445515192	0.0013875803
21	C	11.3868404993	-1.1306034893	0.0443033441
22	H	11.7879692752	-1.6979294733	0.8583251904
23	C	7.2172731291	0.2555106701	0.5317373776

24	H	8.2114841396	0.5771488739	0.7622655722
25	C	11.5671315646	0.4050773318	-1.7239023808
26	H	11.9756719301	1.1234887835	-2.3999590156
27	C	3.6809184636	1.3646256831	-0.0796949444
28	C	2.5885193114	0.9418639344	-0.7566206209
29	O	3.4030559770	3.1225052631	0.6192725186
30	H	2.5532084909	-0.0049504130	-1.2578018831
31	C	1.4817468972	1.8451220543	-0.7875923257
32	C	1.6442993802	3.0226472192	-0.1409814176
33	H	0.5751218703	1.5993892838	-1.3015983342
34	C	0.6770454128	4.0984851502	-0.0267106206
35	C	-0.6727311365	4.0943430361	0.0025182872
36	C	1.1280374520	5.5314543410	0.0262520459
37	C	-1.6352201525	3.0141386141	0.1092866213
38	C	-1.1295652082	5.5260804450	-0.0400792447
39	N	-0.0037178536	6.3152178354	-0.0011107361
40	O	2.2675657555	5.9411219804	0.0983137204
41	O	-3.4139881961	3.1356192268	-0.6015081640
42	C	-1.4564538552	1.8174902900	0.7160054253
43	O	-2.2698923649	5.9338598287	-0.1106466111
44	C	-0.0148181702	7.7683564943	-0.0075464632
45	C	-3.6736969546	1.3578813813	0.0526421624
46	C	-2.5638560326	0.9154936132	0.6876243562
47	H	-0.5372069919	1.5563974407	1.1987161866
48	H	0.9694010216	8.1111379479	0.2665168514
49	H	-0.2670183197	8.1452995905	-0.9885914890
50	H	-0.7394732240	8.1285659959	0.7062218089
51	H	-2.5152348978	-0.0457850214	1.1592824142
52	C	-4.9299567428	0.6610686563	-0.1247799699
53	C	-6.1446905396	1.1943233947	-0.3895201646
54	O	-5.0292233737	-1.2477379150	-0.0468634380
55	H	-6.3031994095	2.2499759390	-0.4856775515
56	C	-7.2234157380	0.2649571106	-0.5080558895
57	C	-6.9152892232	-1.0421954837	-0.3392045965
58	H	-8.2213000262	0.5921843259	-0.7134597719
59	C	-7.8323785281	-2.1610056479	-0.3828500185
60	C	-9.1534322405	-2.2332592271	-0.1312010095
61	C	-7.3729195106	-3.5271151154	-0.8075242564
62	C	-10.0997390697	-1.2778958729	0.4194372904
63	C	-9.6081744789	-3.6286144538	-0.4704729575
64	N	-8.4854708538	-4.3323794461	-0.8588664458
65	O	-6.2365434079	-3.8767096469	-1.0506965528
66	O	-9.7230228810	-0.1917397145	1.9632484017
67	C	-11.3851624126	-1.1221594571	0.0377791017
68	O	-10.7343209465	-4.0750529980	-0.4284579840
69	C	-8.4710894783	-5.7374692057	-1.2289879396
70	C	-11.5274564456	0.3822075867	1.8361150238
71	C	-12.1711727552	-0.1943320169	0.8101142358
72	H	-11.8034994562	-1.6745458760	-0.7778491538
73	H	-7.7799540477	-5.8829567578	-2.0441804112
74	H	-8.1688311898	-6.3543169082	-0.3940603987

75	H	-9.4654521953	-6.0169692364	-1.5368054352
76	H	-11.9211822755	1.0890990165	2.5327908364
77	H	-13.1975843913	0.0080779189	0.5796780563

N-HPPI (**B3LYP-DZ)**

1	C	6.1523336939	-2.4234832853	0.0285085938
2	C	5.4795115968	-1.1567466458	0.0202859680
3	C	7.5592403982	-2.4639279894	-0.0104700364
4	C	6.2336066320	-0.0124842551	-0.0505123485
5	C	5.3908359059	1.0586689501	-0.0682981618
6	H	5.7675596742	2.0569582803	-0.1378525900
7	C	4.0804975788	0.5619822061	0.0097410134
8	C	8.5593765602	0.9547121917	-0.1268962817
9	H	8.3737598762	2.0202770969	-0.1504159600
10	C	7.6272167028	-0.0559602920	-0.0825575869
11	C	8.3016133691	-1.2592845042	-0.0570134337
12	C	7.9235151101	-3.8029271612	0.0069712958
13	C	5.7402819523	-3.7591900548	0.0574528413
14	N	6.8183214207	-4.5930472368	0.0437874450
15	C	6.8074153205	-6.0552892242	0.0553195420
16	H	5.7684245962	-6.4462031467	0.0795297856
17	H	7.3083368727	-6.4387803485	-0.8586646969
18	H	7.3484605485	-6.4261792032	0.9508488571
19	O	9.0567899417	-4.2443803274	-0.0163940638
20	O	4.5949681348	-4.1667977314	0.0744325022
21	N	9.6370480517	-1.0254844013	-0.0836024323
22	N	4.1587710000	-0.8235907033	0.0943362828
23	C	9.7918938390	0.3210626399	-0.1243077132
24	H	10.7493693671	0.8256597219	-0.1482881444
25	C	2.8194470557	1.4461877511	-0.0082862580
26	C	1.5187455769	0.9691416488	-0.1102530386
27	N	2.7593472383	2.8303157995	0.0584166424
28	H	1.1361221753	-0.0168353719	-0.2253092748
29	C	0.6847837484	2.0444942965	-0.0569404899
30	C	1.4292158548	3.1899716831	0.0190985437
31	C	-0.7023912085	2.0501453446	-0.0693023923
32	C	0.7107376994	4.4431264528	0.0301207912
33	C	-1.4460974197	3.1998989790	-0.0250077541
34	C	-1.5360213617	0.9789727516	-0.1015827253
35	C	-0.7212478938	4.4481547975	0.0085425667
36	C	1.0886194522	5.7935663027	0.0432821566
37	N	-2.7796399162	2.8457177136	-0.0262087816
38	H	-1.1512547668	-0.0144066008	-0.1317152421
39	C	-2.8389307627	1.4619609672	-0.0663769186
40	C	-1.0911031557	5.8008283621	0.0219060456
41	N	0.0021828665	6.6012884007	0.0412464526
42	O	2.2131064393	6.2460687682	0.0342323468
43	O	-2.2128906572	6.2580946122	0.0017198534
44	C	0.0233392117	8.0653147061	0.0447540489
45	H	-1.0057804848	8.4822890834	0.0361092423
46	H	0.5445579073	8.4294343677	0.9549278882

47	H	0.5601934537	8.4332648454	-0.8550108433
48	C	-4.0952360874	0.5704474195	-0.0562240536
49	C	-5.4083768870	1.0395300452	-0.0485588339
50	N	-4.1339060849	-0.8150387432	-0.0422535232
51	H	-5.8004110362	2.0354528317	-0.0541665989
52	C	-6.2254857755	-0.0528575012	-0.0320321535
53	C	-5.4559446282	-1.1939704129	-0.0292601846
54	C	-7.6143733139	-0.0913985165	-0.0196453517
55	C	-6.1415192431	-2.4683818960	-0.0146543147
56	C	-8.3135213081	-1.2838306621	-0.0067493089
57	C	-8.5125691700	0.9465244118	-0.0184304927
58	C	-7.5655755324	-2.5011967830	-0.0037158071
59	C	-5.7471895997	-3.8184655953	-0.0099101700
60	N	-9.6527795021	-0.9963306979	-0.0005066193
61	H	-8.2981341256	2.0069148886	-0.0257995703
62	C	-9.7555648965	0.3551184528	-0.0064799249
63	C	-7.9250961658	-3.8485504206	0.0085988063
64	N	-6.8279500182	-4.6390884216	0.0048446868
65	O	-4.6198653863	-4.2601221409	-0.0190294845
66	H	-10.6949343260	0.8945083187	-0.0034535715
67	O	-9.0480625342	-4.3066778213	0.0212509907
68	C	-6.8224664542	-6.1026407942	0.0141553149
69	H	-7.3376443845	-6.4719809177	0.9256129998
70	H	-7.3505028654	-6.4834221187	-0.8851206189
71	H	-5.7856028895	-6.5002930209	0.0094122971
72	C	3.9283622022	3.6986464850	0.1741351957
73	C	-3.9558427760	3.7113251498	0.0257195779
74	C	-2.9509391648	-1.6714769334	-0.0358822845
75	C	-10.8663354855	-1.8140233101	0.0084070203
76	C	3.1123727790	-1.8272100968	0.2959263636
77	C	10.7434914500	-1.9764891459	-0.0660526938
78	H	3.7030406514	4.7352961568	0.3363411459
79	H	4.5215721846	3.6515120401	-0.7621393263
80	H	4.5161088785	3.4131892393	1.0707442900
81	H	-3.7436681187	4.7609976168	0.0863783914
82	H	-4.5389823074	3.4904477417	0.9433365239
83	H	-4.5541078233	3.5813347890	-0.8996244982
84	H	-3.1487636238	-2.7205459213	-0.0014238704
85	H	-2.3540813496	-1.4732405256	0.8786002849
86	H	-2.3797382711	-1.5220468506	-0.9750380634
87	H	-10.6964561117	-2.8830104881	0.0076349429
88	H	-11.4742056753	-1.5801147430	-0.8913490473
89	H	-11.4649928628	-1.5765202086	0.9131900394
90	H	2.1605520582	-1.4379217783	0.6338650471
91	H	3.3821001459	-2.4480143554	1.1736592691
92	H	2.9906765280	-2.4486409776	-0.6161503570
93	H	11.7183569915	-1.4438612700	-0.0875936687
94	H	10.6933011525	-2.6290931138	-0.9616250625
95	H	10.7068826670	-2.5790799927	0.8652819314

N-HPMI (B3LYP-DZ)

1	C	7.5412887079	-2.0399260425	0.3044426209
2	C	6.6503733192	-0.9354312119	0.1921824688
3	C	8.7924008577	-1.9918471880	-0.3300674841
4	C	7.0383355411	0.1768660817	-0.5320878368
5	C	6.0042057560	1.0828701488	-0.5197113332
6	H	5.9757899911	2.0312113627	-1.0398920607
7	C	4.9830367701	0.4732931406	0.2028223049
8	C	8.8703113086	1.1971478553	-1.9320270159
9	H	8.4549643613	2.1581949757	-2.2046350342
10	C	8.2848055781	0.2235664818	-1.1572033441
11	C	9.1637731919	-0.8416278619	-1.0715700702
12	C	9.4166868210	-3.2041284497	-0.0628395266
13	C	7.4702586390	-3.2853942802	0.9243084234
14	N	8.6117626097	-3.9935938979	0.6996355122
15	C	8.9187237848	-5.3433218228	1.1702764503
16	H	8.0797141334	-5.7562155028	1.7697170789
17	H	9.0919667145	-6.0107183616	0.2999517806
18	H	9.8302645063	-5.3207292086	1.8028626568
19	O	10.5186177193	-3.5493318626	-0.4453991270
20	O	6.5380009695	-3.7201212000	1.5727170451
21	N	10.2830235550	-0.5560500632	-1.7800199942
22	N	5.3937902639	-0.7496854880	0.6668813406
23	C	10.1049313925	0.6837442964	-2.3003517978
24	H	10.8285464128	1.1938526777	-2.9232765690
25	C	3.5940051933	1.0133330559	0.3039619518
26	N	3.1534385728	2.3686736669	0.3992134070
27	C	1.3164111070	0.9501115439	0.0954054807
28	C	1.7040820643	2.3699815790	0.3077532452
29	C	-1.3304273409	1.0212825614	-0.1607828711
30	C	0.7742655025	3.4146144112	0.4250844973
31	C	-1.7213521052	2.3919123590	0.2379593387
32	C	-0.7903213027	3.4172911089	0.4353094514
33	C	1.0975738488	4.8528898856	0.6614640797
34	N	-3.1681564874	2.3732810921	0.3512675283
35	C	-3.5980666804	1.0297128311	0.1279400785
36	C	-1.1106412747	4.8418054277	0.7431760867
37	N	-0.0040969520	5.5972405703	0.8451719087
38	O	2.2025841126	5.3573309612	0.6907210947
39	O	-2.2174961100	5.3290246034	0.8576714799
40	C	0.0161781857	7.0273308560	1.1093571349
41	H	-1.0078580328	7.4406918202	1.2266804495
42	H	0.5792545483	7.2270784621	2.0458607548
43	H	0.5105491468	7.5576419837	0.2679746846
44	C	-4.9814887123	0.4731801959	0.0663506108
45	C	-6.0616896724	1.1070970168	-0.5404634419
46	N	-5.3386275629	-0.7837660775	0.4798135043
47	H	-6.0851146149	2.0887862695	-0.9950975642
48	C	-7.0819583693	0.1829979977	-0.5326769019
49	C	-6.6246804641	-0.9633105626	0.0918478406
50	C	-8.3750955054	0.2425341022	-1.0547774496

51	C	-7.4898875129	-2.0869525644	0.2115637022
52	C	-9.2311678557	-0.8412351647	-0.9606359410
53	C	-9.0342292700	1.2475997335	-1.7231071003
54	C	-8.7882442636	-2.0244140188	-0.3167416384
55	C	-7.3550103419	-3.3623343786	0.7556837235
56	N	-10.4076548385	-0.5358085563	-1.5593332327
57	H	-8.6551630979	2.2297442198	-1.9722139970
58	C	-10.2879429335	0.7339325102	-2.0206666682
59	C	-9.3755045206	-3.2588557176	-0.0658160494
60	N	-8.5031474266	-4.0746927053	0.5868164831
61	O	-6.3692852516	-3.8162674172	1.3040255384
62	H	-11.0663257933	1.2653549516	-2.5531936092
63	O	-10.5014129770	-3.5999349448	-0.3751364775
64	C	-8.7564405918	-5.4504573109	1.0118888930
65	H	-9.6130306954	-5.4720981455	1.7172291136
66	H	-8.9932733896	-6.0766914266	0.1262971074
67	H	-7.8670939921	-5.8800095592	1.5203072044
68	C	4.0595653291	3.4675431961	0.6595405294
69	C	-4.0728166590	3.4318970403	0.7476422910
70	C	-4.5092527519	-1.7450927657	1.2131504768
71	C	-11.5995694577	-1.3614413305	-1.7222807940
72	C	4.6389306678	-1.6766431536	1.5153041329
73	C	11.4717249505	-1.3744904587	-1.9939426750
74	H	3.8083155397	3.9370961093	1.6323677561
75	H	4.0450127579	4.1827933482	-0.1880064573
76	H	5.1028701121	3.1256311553	0.7780805962
77	H	-3.7924649555	3.8080839976	1.7528821936
78	H	-5.1103820020	3.0698338144	0.8567576087
79	H	-4.0896910714	4.2273006018	-0.0248266210
80	H	-5.0083808833	-2.0028536027	2.1695131725
81	H	-3.5342299500	-1.3274316934	1.5193091094
82	H	-4.3325807872	-2.6479684430	0.5935336209
83	H	-12.0050718710	-1.6389317835	-0.7277324309
84	H	-11.3499176337	-2.2676514142	-2.3128556617
85	H	-12.3893580983	-0.8060606824	-2.2715978535
86	H	3.6841336979	-1.2511587008	1.8714766733
87	H	5.2141818905	-1.8727314593	2.4432681582
88	H	4.4311373355	-2.6167007220	0.9644396419
89	H	12.2049981169	-0.8406938848	-2.6353887625
90	H	11.1906379961	-2.3150362192	-2.5122419067
91	H	11.9605598163	-1.5911522686	-1.0220729842
92	H	1.0169186334	0.8386522597	-0.9621477005
93	H	-1.0123036675	1.0599436423	-1.2227101317
94	C	2.5703466487	0.2037914713	0.1484863519
95	H	2.6244170017	-0.8613906817	-0.0309637993
96	C	-2.5741697240	0.2576948472	-0.1614801943
97	H	-2.6259291500	-0.7851635148	-0.4446100377

CPPI (B3LYP-DZ)

1	C	14.9348553557	-0.6562398350	-1.3016947807
2	N	13.7176158102	-0.0160410547	-0.8393094804

3	C	12.4453085387	-0.6011127787	-0.9164168202
4	C	11.5049917663	0.3916879020	-0.3362106484
5	C	12.2347669589	1.5070540186	0.0542556750
6	C	11.6083487129	2.6044908446	0.6339298746
7	C	10.2029400653	2.5889054208	0.8289292738
8	C	9.7039099311	3.7934350724	1.4378042907
9	H	8.6525655538	3.9464060658	1.6575190880
10	C	10.6495106836	4.7272653656	1.7185513367
11	H	10.4774285543	5.6935508057	2.1748632498
12	Se	12.3898508312	4.1989793432	1.2321936691
13	C	9.4536415041	1.4436829125	0.4263364059
14	C	8.0418301436	1.2429355476	0.5510482720
15	H	7.3849058026	1.9723259333	1.0124559789
16	C	7.5670074469	0.0449760128	0.0856912339
17	C	6.1910070686	-0.4030169692	0.0811710000
18	S	5.8133794567	-2.1344405338	0.1230207734
19	C	4.0903667312	-1.7891497851	0.0935630016
20	C	3.8443811455	-0.3817288995	0.0758281493
21	C	5.0628095458	0.3719307444	0.0639791594
22	H	5.0823372606	1.4541362247	0.0211040830
23	C	2.5145178394	0.0896698252	0.0556596587
24	C	1.4926090295	-0.8609384303	0.0788524423
25	S	-0.2342045806	-0.5213839321	0.0890503329
26	C	-0.5906754390	-2.2361749889	0.0973674545
27	C	0.5143516966	-3.0237930220	0.0998562404
28	H	0.4893566578	-4.1065050748	0.1225197558
29	C	1.7398470763	-2.2679076440	0.0884709653
30	C	3.0692351713	-2.7387647396	0.0857568773
31	S	3.4481155013	-4.4901097236	0.1128619599
32	C	3.5953762295	-4.8281651904	-1.6887633868
33	H	3.8430827652	-5.8890076305	-1.7824868019
34	H	2.6503294096	-4.6315269325	-2.2004317382
35	H	4.3957657575	-4.2319573837	-2.1326766273
36	S	2.1439285144	1.8428412135	0.0425932996
37	C	1.9041564992	2.1225405402	-1.7592558205
38	H	1.0869138589	1.5076430804	-2.1427331336
39	H	1.6435741242	3.1783441289	-1.8736366714
40	H	2.8234896865	1.9153624567	-2.3118841493
41	Se	8.9573071630	-1.0493517460	-0.6150631717
42	C	10.1282799485	0.3397315501	-0.1618370190
43	C	13.6648173542	1.2642054581	-0.2607124512
44	O	14.6143432947	2.0013052626	-0.0696003630
45	O	12.1971674980	-1.7015760649	-1.3734859681
46	H	15.7589604372	0.0342810566	-1.1155019407
47	H	15.1051573477	-1.5917634807	-0.7605872953
48	H	14.8693721942	-0.8760601810	-2.3714670913
49	C	-2.0612282280	-2.6929289605	0.1185881335
50	C	-3.1521710069	-1.8836986512	0.1156150965
51	Se	-2.4898069914	-4.5258399862	0.1528103375
52	C	-4.4166315061	-2.5701535945	0.1395067721
53	H	-3.0734108413	-0.8018228173	0.0971425199

54	C	-4.2831028844	-3.9826386659	0.1618043514
55	C	-5.7202109583	-1.9907296794	0.1430367181
56	C	-5.4209885345	-4.7810219015	0.1860947781
57	C	-6.0505155304	-0.5979382803	0.1332954550
58	C	-6.8606295086	-2.8383999711	0.1693849060
59	C	-6.6899396534	-4.2163836296	0.1909965905
60	C	-5.5660583842	-6.2580296657	0.2111402285
61	H	-5.2977651334	0.1827809660	0.1506533576
62	C	-7.3884138085	-0.3028592606	0.1420916749
63	Se	-8.4645598980	-1.8721981485	0.1671585535
64	C	-7.6891622517	-5.3151844487	0.2170540832
65	N	-6.9496684471	-6.5068349051	0.2288174238
66	O	-4.6886016472	-7.1015280424	0.2155709551
67	C	-7.9892632400	1.0134762673	0.1237633530
68	O	-8.9035851506	-5.2339105062	0.2258400284
69	C	-7.5434956854	-7.8304705016	0.2549852307
70	S	-9.5971835998	1.2731330740	0.8233003732
71	C	-7.4503707642	2.1681483908	-0.3762330075
72	H	-6.7278033788	-8.5552331828	0.2560730759
73	H	-8.1548487377	-7.9585086683	1.1532381148
74	H	-8.1753493973	-7.9841978861	-0.6248730531
75	C	-9.5363288529	2.9787676966	0.4038083415
76	C	-8.3001761951	3.3123897168	-0.2304470885
77	H	-6.4822454558	2.2147441243	-0.8599173369
78	C	-10.5441967283	3.9126607221	0.6416849996
79	C	-8.0662013105	4.6433098269	-0.6367319262
80	C	-10.3015956203	5.2474177261	0.2562567703
81	S	-12.0702520364	3.4271772956	1.4460203075
82	C	-9.0628176069	5.5837748147	-0.3706997367
83	S	-6.5317756478	5.1234592322	-1.4280040176
84	C	-11.1545930194	6.3983411932	0.4015216104
85	C	-13.0691759493	2.9182182382	-0.0116946109
86	S	-8.9920151183	7.3002609424	-0.7527323201
87	C	-7.0241491130	5.0357063449	-3.1978204987
88	C	-10.5951434648	7.5339712411	-0.0871438366
89	H	-12.1319375214	6.3532332206	0.8664589837
90	H	-14.0345754206	2.5854259392	0.3791793198
91	H	-13.2252200038	3.7602623424	-0.6900725113
92	H	-12.5916555844	2.0907584834	-0.5412517684
93	H	-7.8482737408	5.7214962873	-3.4065069480
94	H	-6.1474416771	5.3413026998	-3.7754902184
95	H	-7.3022609464	4.0163017647	-3.4752415464
96	H	-11.0252396585	8.5274079691	-0.0908647024

CPMI (**B3LYP-DZ)**

1	S	-5.9990023462	2.0896876403	-0.2390251376
2	S	0.0521576205	0.6349083401	-1.4496549955
3	C	-12.6803602497	-1.4603872702	0.4411190629
4	C	-12.2688667075	-2.4469635883	-0.6670719509
5	C	-11.8444822221	-0.7050821204	1.1792828680

6	C	-10.8575778931	-3.5767105012	-2.3481544817
7	H	-10.0023203233	-3.6425332549	-2.9876063246
8	C	-11.7924568707	-4.5694151290	-2.2571807956
9	H	-11.7082631247	-5.5300679637	-2.7204263749
10	C	-8.0023018415	-1.4920635486	0.7238912626
11	H	-7.2609212465	-2.2607999154	0.6598099723
12	C	-10.3082502980	-0.6786046059	1.0653010218
13	C	-7.7180557744	-0.1699823502	0.5222124897
14	C	-6.3626967447	0.3712706921	0.0295366769
15	C	-5.2770101551	-0.4037639375	-0.2550063420
16	H	-5.2882223185	-1.4735447176	-0.2661125799
17	C	-4.0197731747	0.4550249077	-0.5411747658
18	C	-4.2711390775	1.7846115777	-0.4686698697
19	C	-3.1462349957	2.8319654788	-0.5773590351
20	C	-2.6084556768	-0.0718282460	-0.8607283413
21	C	-0.5834260074	3.1507454394	-0.9048540897
22	H	-0.5218172946	4.2139838560	-0.7981179651
23	C	0.5043090824	2.3371254125	-1.1215879683
24	C	-1.9016434706	2.3510977897	-0.8141453116
25	C	-1.6418796986	0.8645828205	-1.0145333943
26	C	-12.6922646015	0.1095991677	2.1702901133
27	C	-14.1163663208	-1.1799279540	0.9137833596
28	N	-14.1225920785	-0.0169617545	1.8242751757
29	C	-14.9931229315	-0.1960654108	2.9958525627
30	H	-16.0055210507	-0.3195810508	2.6726746906
31	H	-14.6843899516	-1.0638092136	3.5403269227
32	H	-14.9229820304	0.6648634872	3.6272943311
33	O	-12.2367302956	0.7780536525	3.1337494336
34	O	-15.1313860016	-1.8448160249	0.5791180870
35	Se	-9.2473300928	0.9167475368	0.9309785422
36	Se	-13.2738027141	-4.0023328860	-1.1746756915
37	S	-3.4665357654	4.5746243487	-0.4020560077
38	S	-2.2707145832	-1.8128018983	-1.0177861124
39	C	-2.2900822151	5.4982813645	-1.3667336331
40	H	-2.4909259166	6.5447029766	-1.2681225032
41	H	-1.3004429350	5.2907805232	-1.0166695857
42	H	-2.3717063711	5.2148564651	-2.3951604063
43	C	-0.9411102780	-2.0646390091	-2.1736569491
44	H	-0.0650752650	-1.5593844975	-1.8245340354
45	H	-0.7380690294	-3.1117382618	-2.2615081884
46	H	-1.2210055205	-1.6742819831	-3.1297581173
47	C	-9.5097534950	-1.7879093477	1.0400708102
48	H	-9.8837280098	-2.7760434581	1.2101486107
49	C	-11.1352360778	-2.3409639023	-1.4232929020
50	H	-10.4859487018	-1.4917630960	-1.3793848131
51	C	1.9604903471	2.8365859094	-1.0806571253
52	C	3.0589669222	2.0514404190	-1.2919228153
53	Se	2.4867380450	4.6472768491	-0.7169996871
54	H	2.9907341925	1.0331948973	-1.6131970720
55	C	4.4267751034	2.7637603763	-1.0069075640
56	C	4.3103939919	4.0601042406	-0.5895677855

57	H	5.3707954659	2.2728347255	-1.1185390316
58	C	5.4773172853	4.9272853143	-0.0825824674
59	C	6.6011679927	4.4762608591	0.5069938851
60	C	5.5549490897	6.4617831548	-0.1415068011
61	C	6.9437775788	3.0021039802	0.7954060843
62	C	7.4717954681	5.6933287064	0.8609565942
63	N	6.9140053379	6.9060803548	0.2287506912
64	O	4.6023376539	7.2232815997	-0.4529446079
65	Se	8.6109334252	2.1580995309	0.3515698865
66	C	6.1013355842	2.1101094411	1.3982559215
67	O	8.5003682025	5.6617264218	1.5845837186
68	C	6.9246566074	8.0822313237	1.1113894100
69	C	7.8348749743	0.4587411036	0.7950399269
70	C	6.6197536064	0.6298569395	1.3980061612
71	H	5.1620400236	2.3957396520	1.8240864472
72	H	6.4735466904	8.9109838657	0.6070888303
73	H	6.3738849517	7.8645100037	2.0024537772
74	H	7.9343412714	8.3271811760	1.3670717633
75	C	8.4868617822	-0.9065433757	0.5059137975
76	H	6.0644241781	-0.1797537021	1.8232672797
77	S	10.0628599467	-1.1256468830	-0.2850219069
78	C	7.9344875783	-2.1115654736	0.8271042087
79	C	10.1080896391	-2.8575581214	0.0751965047
80	H	6.9458236208	-2.2350296843	1.2169505022
81	C	8.9217571333	-3.2788614179	0.5765558787
82	C	11.2949670451	-3.8199688894	-0.1229454592
83	C	8.6613005127	-4.7703350719	0.8579354936
84	C	11.0648083433	-5.1225559717	0.1713390932
85	S	12.8777613096	-3.2502001342	-0.7063060508
86	C	9.6952203049	-5.6125345314	0.6202366088
87	S	7.0899424878	-5.3503024486	1.4614230641
88	C	12.0746209242	-6.2905236618	0.1327155920
89	C	13.6880414943	-4.5549976466	-1.6056044597
90	S	9.7358969784	-7.3705151710	0.7605687084
91	C	6.7946054863	-7.0042320312	0.8743967526
92	H	13.0996302918	-6.1839360529	-0.1565231465
93	C	11.5090592971	-7.4800674770	0.5292560048
94	H	14.6343653819	-4.2070553659	-1.9640284982
95	H	13.8385163583	-5.3939860727	-0.9587326943
96	H	13.0786029674	-4.8481975191	-2.4346233568
97	H	7.5731415205	-7.6501706062	1.2226082713
98	H	5.8516224335	-7.3508102372	1.2432150983
99	H	6.7814042157	-7.0058757532	-0.1954968998
100	H	12.0706729324	-8.3770953380	0.6871992624
